# TBC-8, a Putative RAB-2 GAP, Regulates Dense Core Vesicle Maturation in *Caenorhabditis elegans*

**DOI:** 10.1371/journal.pgen.1002722

**Published:** 2012-05-24

**Authors:** Mandy Hannemann, Nikhil Sasidharan, Jan Hegermann, Lena M. Kutscher, Sabine Koenig, Stefan Eimer

**Affiliations:** 1 European Neuroscience Institute (ENI), Goettingen, Germany; 2 International Max Planck Research School Molecular Biology, Goettingen, Germany; 3 International Max Planck Research School Neuroscience, Goettingen, Germany; 4 DFG Research Center for Molecular Physiology of the Brain (CMPB), Goettingen, Germany; 5 Centre for Biological Signalling Studies (BIOSS), Albert-Ludwigs-Universitaet Freiburg, Freiburg, Germany; Stanford University School of Medicine, United States of America

## Abstract

Dense core vesicles (DCVs) are thought to be generated at the late Golgi apparatus as immature DCVs, which subsequently undergo a maturation process through clathrin-mediated membrane remodeling events. This maturation process is required for efficient processing of neuropeptides within DCVs and for removal of factors that would otherwise interfere with DCV release. Previously, we have shown that the GTPase, RAB-2, and its effector, RIC-19, are involved in DCV maturation in *Caenorhabditis elegans* motoneurons. In *rab-2* mutants, specific cargo is lost from maturing DCVs and missorted into the endosomal/lysosomal degradation route. Cargo loss could be prevented by blocking endosomal delivery. This suggests that RAB-2 is involved in retention of DCV components during the sorting process at the Golgi-endosomal interface. To understand how RAB-2 activity is regulated at the Golgi, we screened for RAB-2–specific GTPase activating proteins (GAPs). We identified a potential RAB-2 GAP, TBC-8, which is exclusively expressed in neurons and which, when depleted, shows similar DCV maturation defects as *rab-2* mutants. We could demonstrate that RAB-2 binds to its putative GAP, TBC-8. Interestingly, TBC-8 also binds to the RAB-2 effector, RIC-19. This interaction appears to be conserved as TBC-8 also interacted with the human ortholog of RIC-19, ICA69. Therefore, we propose that a dynamic ON/OFF cycling of RAB-2 at the Golgi induced by the GAP/effector complex is required for proper DCV maturation.

## Author Summary

Synaptic transmission is mainly mediated by the triggered release of neurotransmitters from synaptic vesicles (SVs). To regulate synaptic transmission and neuronal activity, neurons also release neuropeptides and hormones from dense core vesicles (DCVs). While SVs can be recycled locally at the synapse, DCVs have to be newly synthesized in the cell body after release. The formation of new DCVs requires a multi-step maturation process. During this maturation, the neuropeptides are processed into their active form and factors that would disturb DCV release are removed. Only properly matured DCVs are able to undergo efficient release after stimulation. Since DCV biogenesis mainly uses the normal secretory pathway, an elaborate machinery must exist that guarantees efficient sorting and retention of DCV cargo. Previously, we identified the small GTPase RAB-2 and its effector RIC-19/ICA69 to be involved in the retention of soluble cargo in DCVs. In a screen for molecules that regulate RAB-2 activity during DCV maturation, we identified the evolutionarily conserved TBC domain-containing protein, TBC-8. We demonstrate that TBC-8 is a putative RAB-2 GAP, which also binds to RIC-19/ICA69. Thus, RAB-2 might recruit its own GAP via its effector RIC-19, which suggests that a highly dynamic cycling of RAB-2 is required for DCV maturation.

## Introduction

Most neurons secrete both neurotransmitter filled synaptic vesicles (SVs) as well as dense core vesicles (DCVs) that contain neuropeptides, hormones and trophic factors [1], [2], [3], [4], [5]. The stimulated release of neurotransmitters from SVs mediates fast synaptic transmission, while the release of neuropeptides from DCVs modulates neurotransmission and neuronal activity [2], [3]. Classical neurotransmitters are thought to act locally in the millisecond timescale at their site of release. In contrast, neuropeptides and hormones secreted by DCVs act more slowly and over longer distances [3]. Contrary to SVs that can be recycled locally at the site of release after exocytosis, DCVs have to be synthesized *de novo* in the cell body after release [6]. Despite their importance for the modulation of neurotransmission, neuronal DCV biogenesis is not well understood.

DCVs are generated at the trans-Golgi network (TGN) where neuropeptide precursors and prohormones along with their processing enzymes are sorted and packaged into transport carriers that bud off the Golgi. These immature DCVs (iDCVs) subsequently undergo a maturation process by which processing enzymes, SNAREs (soluble *N*-ethylmaleimide-sensitive factor-attachment protein receptor) that are required for homotypic fusion of iDCVs and lysosomal proteins that have been accidentally packaged into DCV transport carriers are removed by vesicular transport to endosomes [6], [7]. This remodelling of iDCVs is achieved by clathrin mediated sorting at the Golgi-endosomal interface [6], [7], [8], [9]. During their maturation process, iDCVs are continuously acidified by the action of vacuolar ATPases (v-ATPases). This acidification supports the sorting and retention of cargo in iDCVs [6]. It also activates prohormone convertases that proteolytically process prohormones and proneuropeptides into their bio-active forms [10]. The fully processed neuropeptides and hormones subsequently aggregate to a crystalline matrix forming the dense core of DCVs [6], [7], [8], [9]. Mature DCVs (mDCVs) are then transported along microtubules from the cell body to their distal release sites where they are secreted in a regulated manner [11]. Only mDCVs and not iDCVs have been shown to undergo efficient stimulus dependent exocytosis [12], [13].

Two main models have been proposed by which sorting and retention of cargo in forming DCVs could occur: sorting by entry and sorting by retention. The sorting by entry hypothesis predicts the existence of sorting signals and receptors that would actively sort cargo into nascent DCVs [6], [7], [8], [9]. Considering the number of different post-Golgi transport carriers that are generated at the TGN, it is likely that such active sorting mechanisms exist. Furthermore, short N-terminal sequence motifs have been identified in DCV cargos such as provasopressin, pro-oxytocin, pro-opiomelanocortin, and chromogranin A (CgA) and chromogranin B (CgB), which are sufficient for DCV targeting [14], [15], [16], [17]. In contrast, the sorting by retention hypothesis suggests that DCV cargo could passively enter forming DCVs and then be retained during DCV maturation either by active retention in lipid domains or by its aggregation within iDCVs. This model is based on the observations that several DCV factors such as CgA and CgB as well as secretogranin II (SgII) aggregate at pH below 6.5 and high Ca^2+^ concentrations, which occur during iDCV formation at the TGN [17], [18]. Furthermore, it has been shown in the case of secretogranin III (SgIII) that there are direct interactions between aggregated DCV cargos and cholesterol-rich membrane domains of DCVs [18]. These interactions between the different aggregating molecules are likely to generate some sort of higher order retention matrix within iDCVs. In addition, proneuropeptides and prohormones, which enter nascent DCVs in a soluble form, are rendered insoluble once processed by their prohormone convertases. Thus, it is conceivable that after an initial active sorting step to enter nascent DCVs, several DCV core components and processed neuropeptides and hormones are subsequently retained in iDCVs by aggregation during the DCV maturation process.

During sorting of cargo into DCVs, it has been shown that a parallel mechanism exists to remove processing enzymes, lysosomal proteins and mannose-6-phosphate receptors from iDCVs by clathrin dependent sorting processes [19]. In a similar manner, the SNARE proteins syntaxin 6 and VAMP4 as well as synaptotagmin IV are also sorted away from iDCVs and are absent on mDCVs [6], [19], [20]. The fact that during DCV maturation proteins are actively removed suggests that mechanisms must exist to ensure retention of factors that are supposed to stay in mDCVs but are not part of the aggregating core. Therefore, the sorting processes on iDCVs have to be tightly regulated to prevent loss of DCV factors to the endosomal system.

In *Caenorhabditis elegans*, it has recently been shown that RAB-2 is required to retain soluble cargo in maturing DCVs [21], [22]. RAB-2 belongs to the Ras superfamily of small GTPases that act as molecular switches and cycle between an active GTP-bound and an inactive GDP-bound state. Rab GTPases are involved in many steps of vesicular transport including vesicle formation, transport, tethering, docking and fusion of vesicles by binding to effector molecules [23], [24], [25], [26]. In order to regulate the active state of Rab GTPases, two additional enzyme families are required: i) GTPase activating proteins (GAPs) that inactivate Rab proteins by enhancing their low intrinsic GTPase activity, and ii) guanine nucleotide exchange factors (GEFs) that activate Rab GTPases by facilitating the exchange of GDP with GTP [27]. Most of the known Rab GAPs possess a conserved Tre2/Bub2/Cdc16 (TBC) domain (except for Rab3A-GAP [28]) consisting of approximately 200 amino acids. This TBC-domain is sufficient for GAP activity *in vitro* by employing an arginine finger-based catalytic mechanism [29]. Interestingly, some TBC proteins are modular and contain additional domains that are important mainly for the regulation of protein-protein interactions or for targeting these proteins to membranes, suggesting additional role(s) and/or regulation of these proteins [30].

In order to understand the spatio-temporal regulation of RAB-2 during DCV maturation, we aimed to identify a GAP for RAB-2 in *C. elegans*. By using a DCV trafficking assay for neuronal TBC domain-containing proteins in *C. elegans*, we were able to identify the evolutionarily conserved protein, TBC-8, as a putative, neuron specific RAB-2 GAP (GenBank: CAA84706.3). As in *rab-2* mutants, loss of TBC-8 showed a similar DCV trafficking phenotype, whereas the transport of SVs is not affected. Furthermore, we could demonstrate that TBC-8 binds to the active form of RAB-2. Lastly, overexpression of TBC-8 in neurons redistributed RAB-2 from Golgi membranes to the cytosol. Taken together our *in vivo* data suggest that TBC-8 is a putative RAB-2 GAP. Unfortunately, we were unable to obtain soluble full-length or fragments of TBC-8 to show GAP activity towards RAB-2 or other RABs *in vitro*. Interestingly, we could show that TBC-8 also binds to the RAB-2 effector, RIC-19/ICA69, indicating that RIC-19 might recruit the GAP to RAB-2 positive membranes to inactivate this small GTPase. These results suggest that a dynamic cycling of RAB-2 at the Golgi is necessary for proper DCV maturation.

## Results

### *tbc-8* mutants display DCV maturation defects

**Figure 1:**
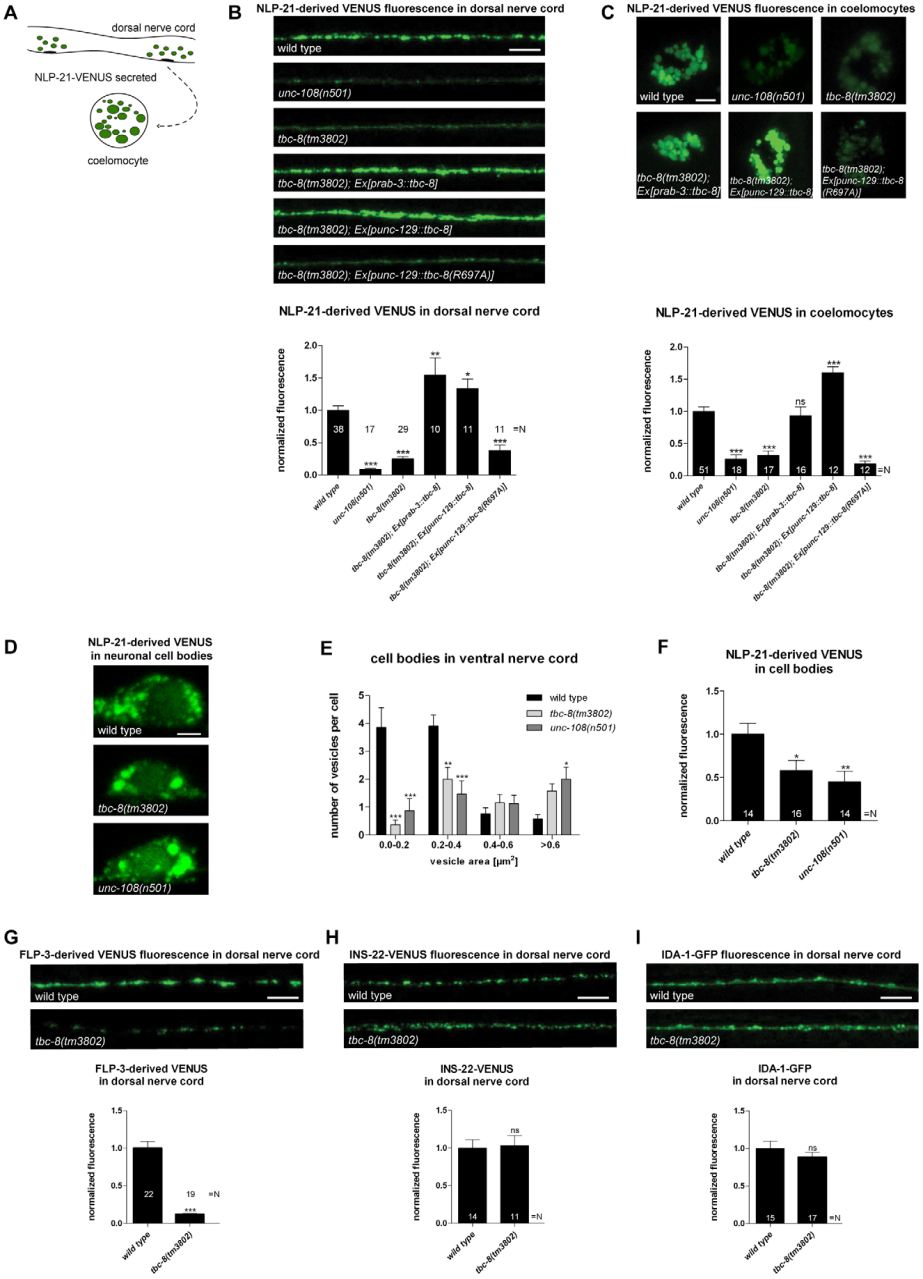
*tbc-8(tm3802)* mutant shows defects in DCV maturation. (A) Schematic representation of DCV assay used in this study. The proneuropeptide NLP-21-VENUS fusion protein is expressed in dorsally projecting DA and DB cholinergic motoneurons. VENUS labeled DCVs are transported to the DNC where VENUS is secreted into the body cavity. Here, VENUS is taken up by scavenger cells of *C. elegans* (called coelomocytes) [31], [32]. *tbc-8(tm3802)* showed decreased NLP-21-derived VENUS levels in the DNC (B) and in the coelomocytes (C), similar to *unc-108/rab-2(n501)* mutants. NLP-21-derived VENUS levels were rescued by expressing *tbc-8* pan-neuronally (*rab-3* promoter) as well as specifically in DA and DB cholinergic motoneurons (*unc-129* promoter) demonstrating that TBC-8 acts cell autonomously. Strikingly, the catalytically inactive TBC-8 (R697A) mutant was not able to rescue the DCV phenotype, emphasizing that its GAP activity is responsible for decreased NLP-21-derived VENUS levels at the axons and in coelomocytes. (D) Representative pictures of NLP-21-derived VENUS fluorescence in the cell bodies of cholinergic motoneurons in the ventral nerve cord of wild type worms, *tbc-8(tm3802)* and *unc-108(n501)* mutants are shown. Scale bar represents 2 µm. Size distribution of VENUS positive vesicular structures in these mutants were analyzed in (E). Error bars = s.e.m (N = 15–21). (***, P<0.0001; **, P<0.001, ANOVA with Bonferroni post test). (F) *tbc-8(tm3802)* and *unc-108(n501)* mutants showed decreased fluorescence intensities of NLP-21-derived VENUS in cell bodies of cholinergic motoneurons in the ventral nerve cord compared to wild type worms. (G) The FMRFamide related peptide FLP-3-derived VENUS fluorescence was also decreased in *tbc-8(tm3802)*. (H) Analysis of VENUS-tagged insulin-like neuropeptide, INS-22, was not changed in fluorescence intensities in *tbc-8(tm3802)*. (I) The fluorescence level of the transmembrane protein, IDA-1-VENUS, was not affected in *tbc-8(tm3802)*. Scale bars in DNC and coelomocytes represent 5 µm. Error bars = s.e.m. (***, P<0.0001; **, P<0.001; *, P<0.05, Student's t-test).

To identify the Rab GAP that regulates RAB-2 during neuronal DCV maturation, we systematically tested mutants of TBC domain-containing GAPs in *C. elegans*. These strains were analyzed for trafficking defects of the DCV marker NLP-21-VENUS (Figure S1). NLP-21-VENUS is a fusion protein of the proneuropeptide NLP-21 with the yellow fluorescent protein VENUS. In this assay, we used an integrated strain stably expressing fluorescently-labeled neuropeptide, NLP-21-VENUS, specifically in DA and DB cholinergic motoneurons in *C. elegans*. It was shown that NLP-21-VENUS is packaged into DCVs, transported to axons of the dorsal nerve cord (DNC), and released into the body cavity [31]. Once secreted, NLP-21-derived VENUS is subsequently endocytosed by six macrophage-like scavenger cells (called coelomocytes), which constantly filter the body fluid by bulk endocytosis [32] (Figure 1A).

In this screen, only *tbc-8(tm3802)* deletion mutants displayed decreased NLP-21-derived VENUS levels in the DNC axons, similar to *unc-108/rab-2* mutants [21], [22] (Figure S1). Inactivation of TBC-8 led to a 74.95±3.51% decrease of NLP-21-derived VENUS fluorescence in axons compared to wild type (Figure 1B). We observed comparable results when *tbc-8* expression was downregulated by RNAi (Figure S2). Similar to *unc-108/rab-2* mutants, the accumulation of secreted VENUS in coelomocytes was also decreased by 68.17±6.94% (Figure 1C) suggesting that less VENUS was secreted. To determine whether the observed DCV phenotype was caused by TBC-8 malfunction in the nervous system, we expressed *tbc-8* cDNA pan-neuronally under the control of the *rab-3* promoter. Pan-neuronal expression of *tbc-8* as well as expression of *tbc-8* under the DA and DB cholinergic motoneuron specific *unc-129* promoter (2641 bp promoter fragment upstream of the start ATG of *unc-129*) was able to rescue the NLP-21-derived VENUS fluorescence levels in DNC and in coelomocytes (Figure 1B, 1C). This showed that TBC-8 is cell-autonomously required in cholinergic motoneurons for proper DCV function. The catalytic GAP activity of TBC-8 was also shown to be indispensible for DCV trafficking as a catalytically inactive TBC-8 (R697A) was unable to rescue the loss of NLP-21-derived VENUS fluorescence (Figure 1B, 1C). This result demonstrates that TBC-8 acts as a RAB GAP required for effective DCV function.

Previously, it was shown that in *unc-108/rab-2* mutants less NLP-21-VENUS-derived cargo is present in mature DCVs, because the cargo was partially lost in the endosomal-lysosomal degradation route during DCV maturation [21], [22]. These trafficking defects resulted in aberrant NLP-21-derived VENUS accumulation in the cell bodies of *unc-108(n501)* mutants. In order to test whether *tbc-8(tm3802)* mutants displayed the same trafficking defects, we analyzed the size distribution of VENUS positive vesicular structures in the cell bodies of these mutants. Similar to *unc-108(n501)* mutants, *tbc-8(tm3802)* mutant worms contained more of these large VENUS positive vesicular structures in the neuronal cell bodies, whereas smaller vesicular structures filled with NLP-21-derived VENUS are less present compared to wild type animals (Figure 1D, 1E). Furthermore, the overall fluorescence of NLP-21-derived VENUS in the cell bodies of motoneurons is decreased in *tbc-8(tm3802)* mutants (58.23±11.71%) like in *unc-108(n501)* worms (45.16±18.98%) (Figure 1F). These results indicate that *tbc-8(tm3802)* mutants have comparable DCV trafficking defects similar to *unc-108/rab-2* mutants.

To determine whether neuropeptides other than NLP-21 are also affected in *tbc-8(tm3802)*, we tested the fluorescence levels of FMRFamide related peptide FLP-3-derived VENUS and the insulin-like neuropeptide INS-22-VENUS in the DNC (Figure 1G, 1H). FLP-3-derived VENUS levels in the DNC of *tbc-8* mutants were decreased by 87.90±1.19%, suggesting that VENUS derived from other neuropeptides was also lost. However, INS-22-derived VENUS fluorescence levels were unchanged in *tbc-8(tm3802)*, like in *unc-108/rab-2* mutants [21], [22]. In addition to different neuropeptides, we also tested a transmembrane protein of DCVs, IDA-1-GFP, in the DNC. IDA-1-GFP levels were unchanged in the *tbc-8* background when compared to wild type animals, suggesting no changes in the numbers of DCVs in *tbc-8* mutants (Figure 1I). This observation was supported by a high-pressure freeze electron microscopy (HPF-EM) analysis of the DCV numbers and distribution at axonal release sites in *tbc-8* mutants, which were unaltered compared to wild type (Table 1). Therefore, in *tbc-8* mutants, DCVs are loaded with less soluble NLP-21-derived VENUS, as it has also been shown for *unc-108/rab-2* mutants [21], [22]. Thus, DCV maturation is disrupted in *tbc-8* mutants in a manner similar to *unc-108/rab-2* mutants.

**Table 1:** SV and DCV analysis determined by HPF EM.

Genotype	Number of profiles/animals analyzed	Mean area of presynaptic terminal in cross sections [µm^2^]	SVs/profile	Mean diameter of SVs [nm] (number of analyzed SVs)	DCVs/profile	Mean diameter of DCVs [nm] (number of analyzed DCVs)
wild type	79/8	0.21±0.01	33.7±1.2	29.6±0.7 (89)	2.1±0.2	43.6±0.8 (49)
*tbc-8(tm3802)*	48/3	0.19±0.01	37.3±1.5	30.0±0.4 (41)	2.0±0.4	41.5±0.7 (77) ns
*unc-108(n501)*	25/3	0.24±0.04	38.2±1.9	27.8±0.7 (97)	2.3±0.3	49.2±1.6 (59) **

Only significant differences of mutant strains compared to wild type worms are indicated. Mean ± s.e.m. are shown (**, P<0.001; Student's t-test). The mean diameter of DCVs in *unc-108(n501)* mutants was more viable, which was shown previously [22].

### TBC-8 and RAB-2 are involved in the same genetic pathway

**Figure 2:**
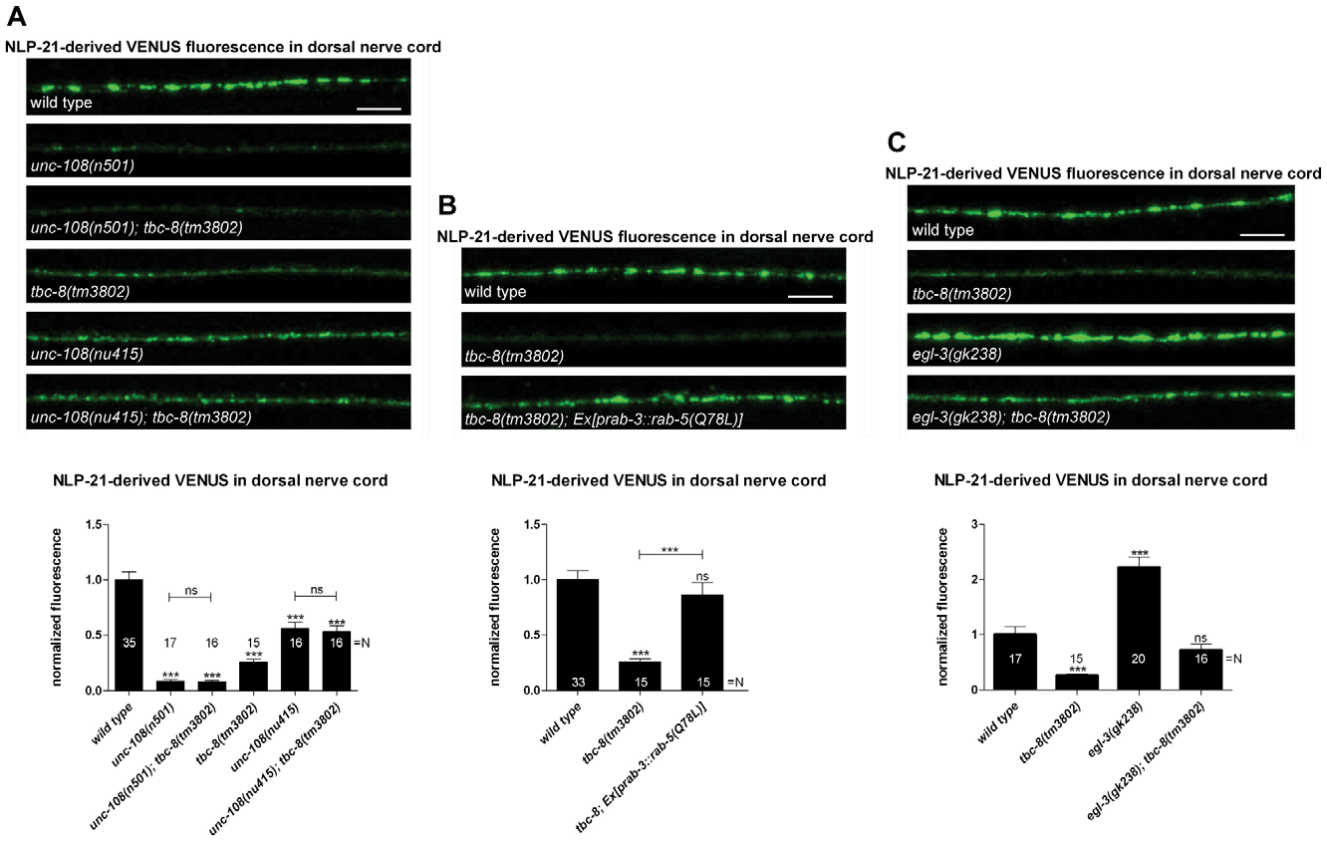
TBC-8 is involved in the same pathway as UNC-108/RAB-2. (A) Double mutants of *tbc-8(tm3802)* and dominant active *unc-108/rab-2(n501)* or null allele *unc-108/rab-2(nu415)*, respectively, followed the phenotype of the *unc-108/rab-2* single mutants. (B) Interfering with the endosomal/lysosomal pathway by overexpression of constitutively GTP-bound RAB-5 (Q78L) in a *tbc-8(tm3802)* mutant background restored the NLP-21-derived VENUS fluorescence at the DNC back to wild type level. (C) TBC-8 is not involved in the generation of active NLP-21 neuropeptides. The double mutant *egl-3(gk238); tbc-8(tm3802)* restored NLP-21-derived VENUS cargo in the DNC back to wild type levels. Scale bar represents 5 µm. Error bars = s.e.m. (***, P<0.0001; Student's t-test).

In order to see whether TBC-8 acts in the same genetic pathway as RAB-2, we compared *tbc-8(tm3802)* with different *unc-108/rab-2* mutants. We used two different alleles of RAB-2, *(n501)* D122N, which is dominant active and constitutively bound to GTP [22], and the deletion *(nu415)* that serves as a molecular null allele since the protein product could not be detected on Western blots [33]. In *unc-108/rab-2(n501)* NLP-21-derived VENUS fluorescence levels in the DNC are more strongly decreased as compared to *(nu415)* by 91.13±1.38% and 43.89±5.59%, respectively (Figure 2A). If TBC-8 is the GAP for RAB-2, then in *tbc-8* mutants, RAB-2 should predominantly be in the constitutively GTP-bound form and therefore, *tbc-8* mutants should resemble the dominant *unc-108/rab-2(n501)* mutant. Consistent with this idea, the *tbc-8(tm3802)* deletion allele indeed showed a decrease in NLP-21-derived VENUS fluorescence (74.00±2.51%), similar to *unc-108(n501)* mutants (91.13±1.38%) (Figure 2A). Comparatively, the decrease in NLP-21-derived VENUS fluorescence was not as pronounced in the *unc-108(nu415)* null allele (43.89±5.59%) (Figure 2A). This result suggests that RAB-2 is indeed in the active GTP-bound form in *tbc-8* mutants. Therefore, inactivation of RAB-2 in a *tbc-8* mutant background should lead to a weaker NLP-21-VENUS phenotype, identical to the *unc-108/rab-2(nu415)* null allele. In agreement with this hypothesis, *unc-108(nu415); tbc-8(tm3802)*, a combination of both deletion alleles, showed an identical phenotype (46.88±5.20%) as the single *unc-108(nu415)* deletion allele (43.89±5.59%) (Figure 2A). Furthermore, *unc-108(n501); tbc-8(tm3802)*, a double mutant with *unc-108/rab-2* gain of function allele, (91.84±1.37%) also resembled the single *unc-108(n501)* allele (91.13±1.38%) (Figure 2A). Both double mutants followed the phenotype of the respective *unc-108* single mutant indicating that both proteins are involved in the same genetic pathway.

Previously, we have shown that the expression of a constitutively GTP-bound form of RAB-5 (Q78L) could rescue the DCV phenotype in the *unc-108(n501)* mutant by inhibiting loss of DCV cargos to early endosomes and returned NLP-21-derived VENUS fluorescence levels back to wild type levels [22]. Since TBC-8 and RAB-2 are in the same pathway, RAB-5 (Q78L) should also rescue the NLP-21-VENUS phenotype in the *tbc-8(tm3802)* deletion allele. Consistent with this hypothesis, expression of RAB-5 (Q78L) could rescue the NLP-21-derived VENUS fluorescence levels at the synapses up to 86.47±10.90%, as seen in Figure 2B. This result indicates that TBC-8, like RAB-2, is required for retaining specific cargo in maturing DCVs by preventing its entry to the late endosomal system.

In order to test whether TBC-8 is involved in neuropeptide processing, we generated a double mutant of the prohormone convertase PC2, *egl-3(gk238)*, and *tbc-8(tm3802)*, and tested it for defects in DCV maturation (Figure 2C). The data was compared with the respective single mutants to identify possible genetic interactions (Figure 2C). Previously, it has been shown that the loss of NLP-21-derived VENUS from maturing DCVs in *unc-108/rab-2* mutants can be rescued by inactivation of EGL-3 [22]. Thus, the lack of proneuropeptide processing retains NLP-21-VENUS into the insoluble dense core of DCVs of *egl-3(gk238)*; *unc-108/rab-2* mutants. Similarly, in *egl-3(gk238)*; *tbc-8(tm3802)* double mutants, NLP-21-derived VENUS levels were restored to wild type levels (72.50±9.90%) (Figure 2C). This finding indicates that primarily soluble NLP-21-derived VENUS is lost in *unc-108/rab-2* and *tbc-8(tm3802)* mutants. Although *unc-108/rab-2; egl-3(gk238)* double mutants had restored levels of NLP-21-derived VENUS in the DNC, analysis of the movement phenotype in the double mutants revealed that they were more severely uncoordinated than either single mutant [22]. This result suggests that, despite rescuing the NLP-21-derived VENUS marker in the DNC, the combined loss of soluble cargo and functional neuropeptides in DCVs leads to additive locomotion defects in *unc-108/rab-2; egl-3(gk238)* double mutants. Interestingly, *tbc-8(tm3802)* mutants do not display a movement defect (Figure S3) and double mutants of *tbc-8(tm3802); egl-3(gk238)* do not yield animals that are similarly uncoordinated to *unc-108/rab-2; egl-3(gk238)* mutants (data not shown; [22]). These data suggest that RAB-2's role in regulating movement may predominantly occur in a subset of neurons that do not express *tbc-8*, but perhaps express another GAP.

All these findings highlight that TBC-8 is indeed in the same pathway as RAB-2 and thus, TBC-8 is a likely candidate to be a RAB-2 GAP. However, differences in movement defects between *tbc-8* and *unc-108/rab-2* mutants suggest the existence of additional GAPs, which may regulate RAB-2 activity in the absence of TBC-8.

### *tbc-8* mutants show no morphological defects in motoneurons and no SV defects

**Figure 3:**
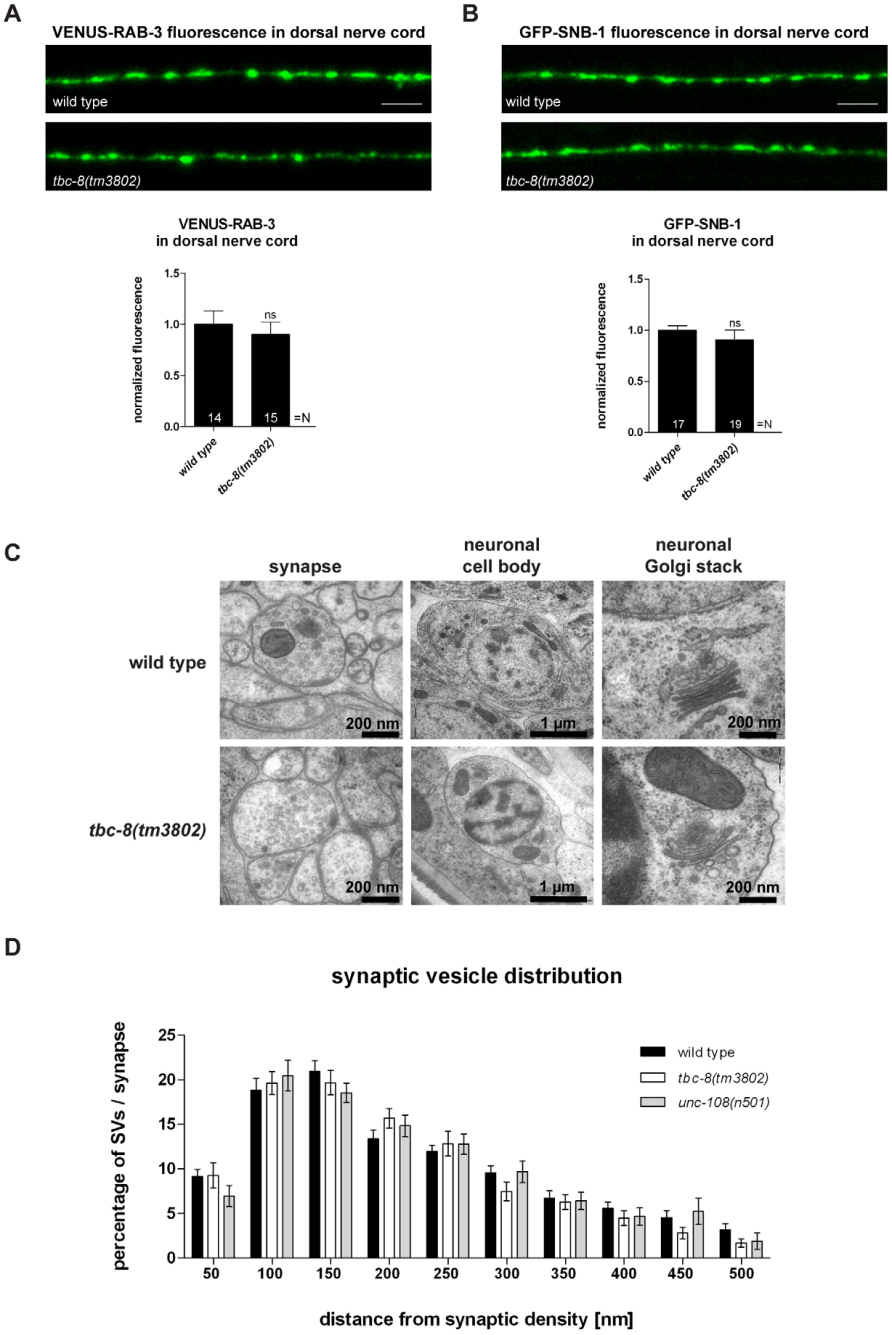
*tbc-8(tm3802)* mutants display no defects in synaptic vesicle trafficking and localization. The transport of synaptic vesicles to the axonal release sites in the DNC was visualized by VENUS-tagged RAB-3 (VENUS-RAB-3) (A) and GFP-tagged synaptobrevin (GFP-SNB-1) (B). No differences were observed in transport of both SV markers in *tbc-8(tm3802)* mutants. Scale bar represents 5 µm. Error bars = s.e.m. (ns, P>0.05; Student's t-test). (C) Electron microscopic analysis of *tbc-8(tm3802)* mutants revealed no obvious differences in the morphology of synapses, cell bodies and neuronal Golgi stacks compared to wild type. (D) The synaptic vesicle distribution relative to the presynaptic density at synapses of *tbc-8(tm3802)* mutants was similar to wild type worms (Student's t-test).

For *unc-108/rab-2* mutants, it has been shown that DCV maturation is specifically affected while SV trafficking and function are unaltered [21], [22]. To exclude that the lack of TBC-8 would lead to general membrane trafficking defects in neurons, we analyzed SV localization, distribution and morphology. We first examined synaptic morphology by analyzing the localization of the synaptic marker proteins, RAB-3 and Synaptobrevin-1 (SNB-1), using integrated strains expressing YFP-RAB-3 and GFP-SNB-1 in cholinergic motoneurons. The fluorescence of YFP-RAB-3 (90.12±11.96%) (Figure 3A) and GFP-SNB-1 (90.82±9.32%) (Figure 3B) was unchanged in the DNC of *tbc-8(tm3802)* when compared to wild type. Second, we used HPF-EM to show that there were no obvious changes in the morphology of synapses, neuronal cell bodies and neuronal Golgi complexes in *tbc-8(tm3802)* mutants compared to wild type worms (Figure 3C). Here, we did not detect any abnormal accumulation of vesicles or degeneration of the Golgi stacks in *tbc-8(tm3802)* mutants compared to wild type animals.

To exclude that there are more subtle changes in SV distribution at presynaptic active zones in *tbc-8(tm3802)* mutants, we analyzed SV distributions surrounding the presynaptic dense projection of cholinergic motoneurons by HPF-EM. There were no changes in SV distribution, SV numbers or SV diameter as compared to wild type (Figure 3D, Table 1). This analysis demonstrates that SV function is unaffected in *tbc-8(tm3802)* mutants, similar to what has been reported for *unc-108/rab-2* mutants [21], [22]. For *unc-108/rab-2* gain of function mutants, it has been shown that DCV diameters are more variable [21], [22] (Table 1). However, this was not the case for *tbc-8(tm3802)*, where DCV diameters are not significantly different from wild type DCVs (Table 1).

### *tbc-8* codes for an evolutionarily conserved Rab GAP specifically expressed in neurons

**Figure 4:**
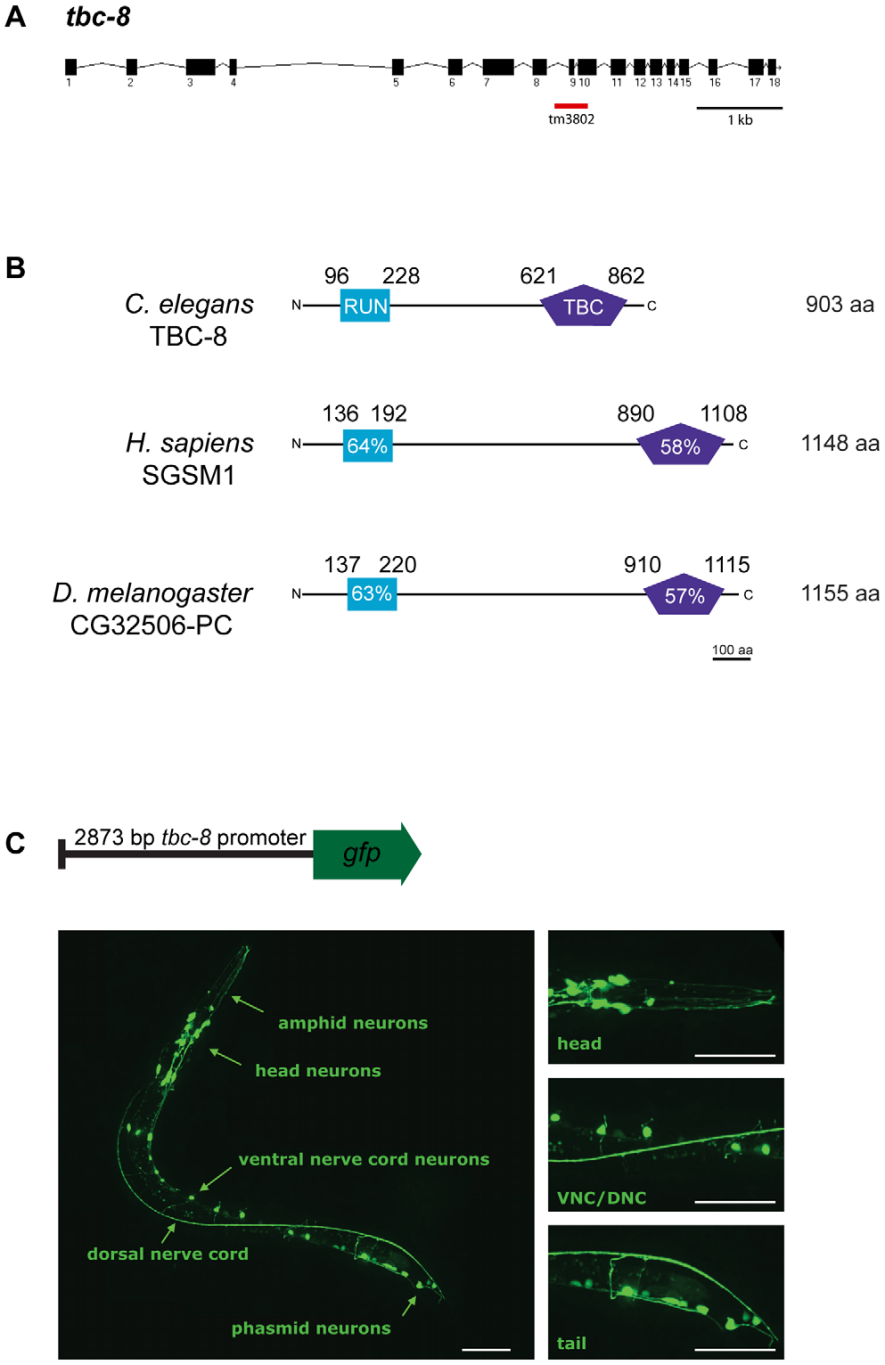
TBC-8 is exclusively expressed in neurons. (A) Gene structure of *tbc-8*. Exons are depicted as black boxes and introns as black lines. The position of the *tm3802* deletion is indicated (red line). (B) TBC-8 is evolutionarily conserved. Orthologs of TBC-8 are found in *H. sapiens* and *D. melanogaster*. TBC-8 contains two predicted domains, a RUN domain (blue) and a TBC-domain (purple). Percentage similarities for both domains are shown relative to *C. elegans* TBC-8. For full protein sequences of TBC-8 and its orthologs, see Figure S4. (C) Schematic representation of a construct containing a 2873 bp *tbc-8* promoter fused to *gfp*. This transcriptional reporter construct was injected into wild type worms and *gfp* expression was imaged in stage three larval (L3) worms by confocal microscopy. *tbc-8* expression was observed in the neurons of the head including amphid neurons, the ventral nerve cord (VNC) neurons and in neurons of the tail (phasmid neurons). The right panel shows magnifications of the respective regions. Scale bars represent 50 µm.

*tbc-8* encodes a 903 amino acid (aa) protein. The *tbc-8(tm3802)* deletion allele leads to a stop codon after the 8^th^ exon, truncating the protein after aa 482 just before the TBC-domain (Figure 4A). The TBC-domain of TBC-8 is located at the C-terminus between aa 621 and 862. In addition to the TBC-domain, TBC-8 also contains a conserved RUN [after RPIP8 (RaP2 interacting protein 8)/UNC-14/NESCA(new molecule containing SH3 at the carboxyl-terminus)] domain (Figure 4B), which has been shown to bind to small GTPases of the Rab and Rap family [34], [35]. TBC-domain proteins with the same domain structure can be found in *Drosophila melanogaster* and the mammalian system [30] (Figure 4B). The orthologs in *D. melanogaster* (CG32506-PC, FlyBase ID: FBpp0300194) and in *H. sapiens* (SGSM1 [36], accession number: NP_001035037) are about 250 aa longer than *C. elegans* TBC-8. The RUN domains of these proteins show a 64% (SGSM1) and a 63% (CG32506-PC) similarity to the RUN domain of TBC-8, whereas both TBC-domains are about 58% (SGSM1) and 57% (CG32506-PC) similar to the TBC-domain of *C. elegans* TBC-8. For full protein sequences of TBC-8 and its orthologs see Figure S4.

In order to determine the expression pattern of *tbc-8*, a construct containing a 2873 bp genomic fragment of the *tbc-8* promoter region was fused to *gfp* and injected into wild type worms (Figure 4C, upper panel). The expression pattern using a transcriptional reporter revealed that *tbc-8* is exclusively expressed in neurons including the head and tail neurons as well as neurons in the ventral nerve cord (VNC) (Figure 4C, lower panel). Interestingly, analysis of the expression pattern of *unc-108/rab-2* showed a similar high expression in neurons [21], [22].

### TBC-8 shows no defects in postendocytic trafficking and is not required for degradation of apoptotic cell corpses

Based on our expression analysis, we could show that *tbc-8* is expressed in the nervous system (Figure 4C). To exclude the possibility that our transcriptional reporter missed important regulatory regions and elements, we tested TBC-8 function in other tissues in which RAB-2 activity was shown to be required [33], [37], [38]. We analyzed the possible role of TBC-8 in postendocytic trafficking in coelomocytes (Figure S5) and in the degradation of apoptotic cell corpses in the germ line (Figure S6).

We did not detect any defects in coelomocytes during the steady-state endocytosis of ssGFP from muscle cells (*arIs37[*p*myo-3::ssGFP]*) [39] in *tbc-8(tm3802)* mutants (Figure S5A).

In order to test the kinetics of ssGFP uptake and the degradation of endocytosed GFP in coelomocytes, we used a strain that expresses ssGFP under a heat-shock promoter (*arIs36[*p*hsp::ssGFP]* [39]) which allowed us to perform an *in-vivo* pulse-chase experiment in *C. elegans*. While following the fate of secreted GFP within coelomocytes after a short heat-shock, no defects were observed in *tbc-8(tm3802)* mutants compared to wild type worms for all measured time points (Figure S5B).

In addition, we examined postendocytic trafficking of the fluid-phase marker Texas red-BSA (TR-BSA) in coelomocytes of *tbc-8(tm3802)* mutants. TR-BSA was microinjected into the pseudocoelom of young adult worms. Using a marker for endosomes, RME-8-GFP, we analyzed the migration of injected TR-BSA through RME-8-GFP positive compartments into later postendocytic compartments (Figure S5C). We did not see obvious defects at any time point in *tbc-8(tm3802)* mutants compared to wild type animals.

Previously, it was shown that RAB-2 is also required for the degradation of apoptotic cell corpses in *C. elegans* [37], [38]. To identify whether TBC-8 plays a role together with RAB-2 in the germ line, we assayed the presence of apoptotic cell corpses in *tbc-8(tm3802)* mutants (Figure S6). Unlike *unc-108/rab-2* deletion mutants, *tbc-8(tm3802)* mutants showed similar numbers of apoptotic cell corpses as wild type worms (Figure S6).

These data together with the expression analysis indicate that TBC-8 likely regulates RAB-2 function only in the nervous system of *C. elegans*.

### TBC-8 localizes to the Golgi-endosomal interface

**Figure 5:**
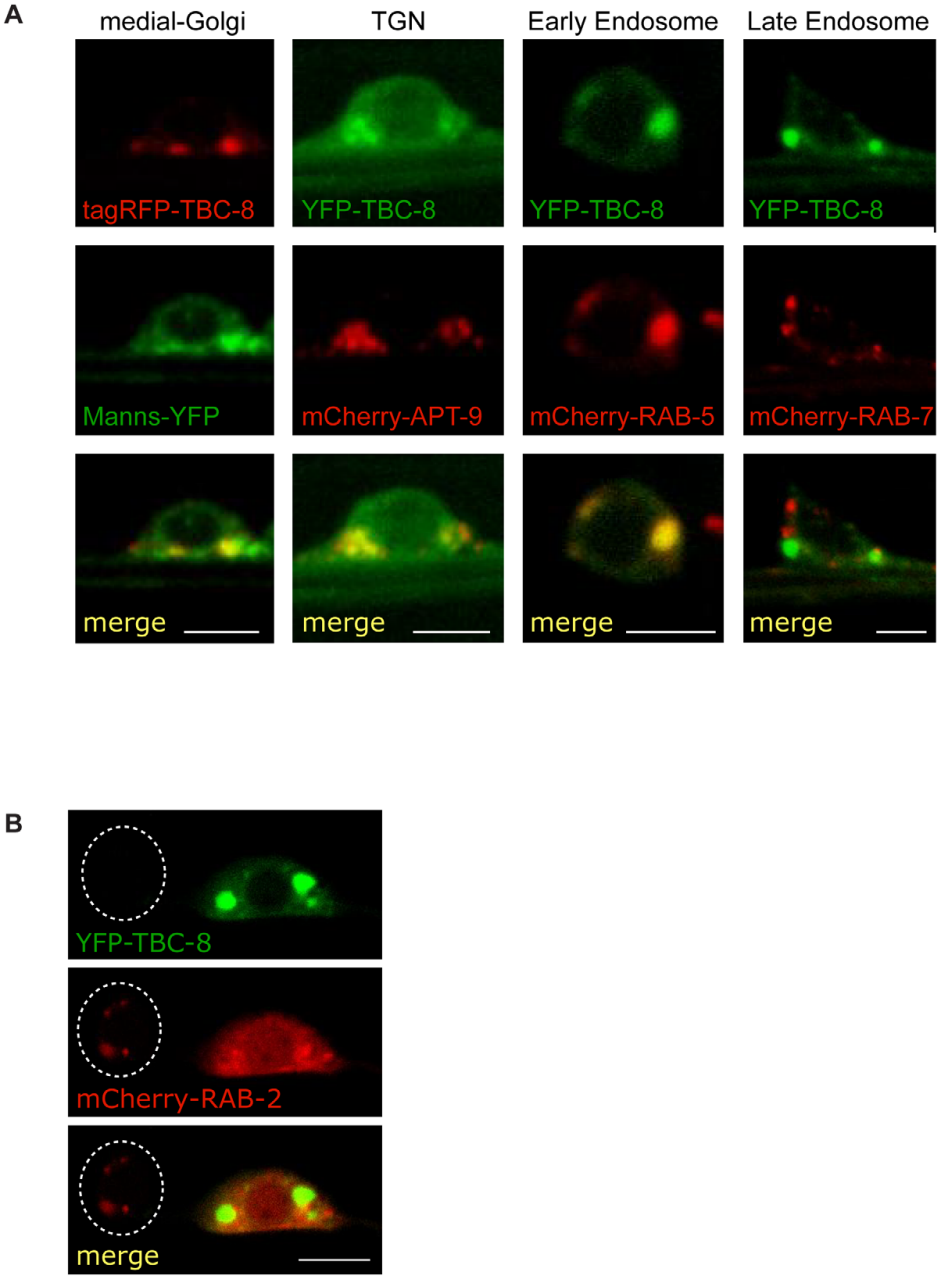
TBC-8 localizes to the Golgi-endosomal interface. (A) The subcellular localization of fluorescently-labeled TBC-8 expressed in motoneurons was analyzed by confocal microscopy. Fluorescently-labeled-TBC-8 localized partially with medial and trans-Golgi markers and showed full co-localization with the early endosomal marker mCherry-RAB-5. No co-localization to RAB-7-positive late endosomes was observed. (B) In motoneurons, mCherry-RAB-2 localizes to the Golgi (left cell body; cell boundary is highlighted by a dashed line). However, when YFP-TBC-8 is overexpressed, mCherry-RAB-2 is redistributed to the cytosol (right cell body). Manns: mannosidase II, TGN: trans-Golgi network. Scale bars represent 3 µm.

In order to examine where TBC-8 functions, we expressed fluorescently labeled TBC-8 under the *rab-3* pan-neuronal promoter and determined its sub-cellular localization in motoneurons. Fluorescently labeled TBC-8 showed a cytosolic localization with some punctate membrane staining (Figure 5A) reminiscent of RAB-2. In order to identify which subcellular compartments these cytoplasmic puncta correspond to, we co-localized fluorescently-labeled TBC-8 with different subcellular markers in neurons (Figure 5A). We observed partial localization of TBC-8 with medial (mannosidase II) and trans-Golgi (APT-9) markers and an almost complete co-localization with the early endosomal marker RAB-5. However, we could not observe any localization of TBC-8 to RAB-7-positive late endosomes. This suggests that TBC-8 acts at the Golgi-endosomal interface, similar to RAB-2, where DCV maturation is also believed to take place [7].

### TBC-8 is a putative RAB-2 GAP

RAB-2 was shown to localize to the Golgi [22]. Attempts to co-localize TBC-8 and RAB-2 were difficult, because when YFP-TBC-8 fusion proteins were co-expressed with mCherry-RAB-2 in motoneurons, we observed that RAB-2 was mainly cytosolic (Figure 5B, right cell body). However, in neurons where YFP-TBC-8 was not expressed due to the mosaic nature of extra-chromosomal arrays in *C. elegans*, a distinct Golgi staining of mCherry-RAB-2 was visible in these cell bodies (Figure 5B, left cell body). This is a strong indication that TBC-8, when overexpressed, inactivates RAB-2, which in turn would be redistributed from the Golgi to the cytosol. This result reinforces the idea that TBC-8 is a RAB-2 GAP as evident from the genetic data.

**Figure 6:**
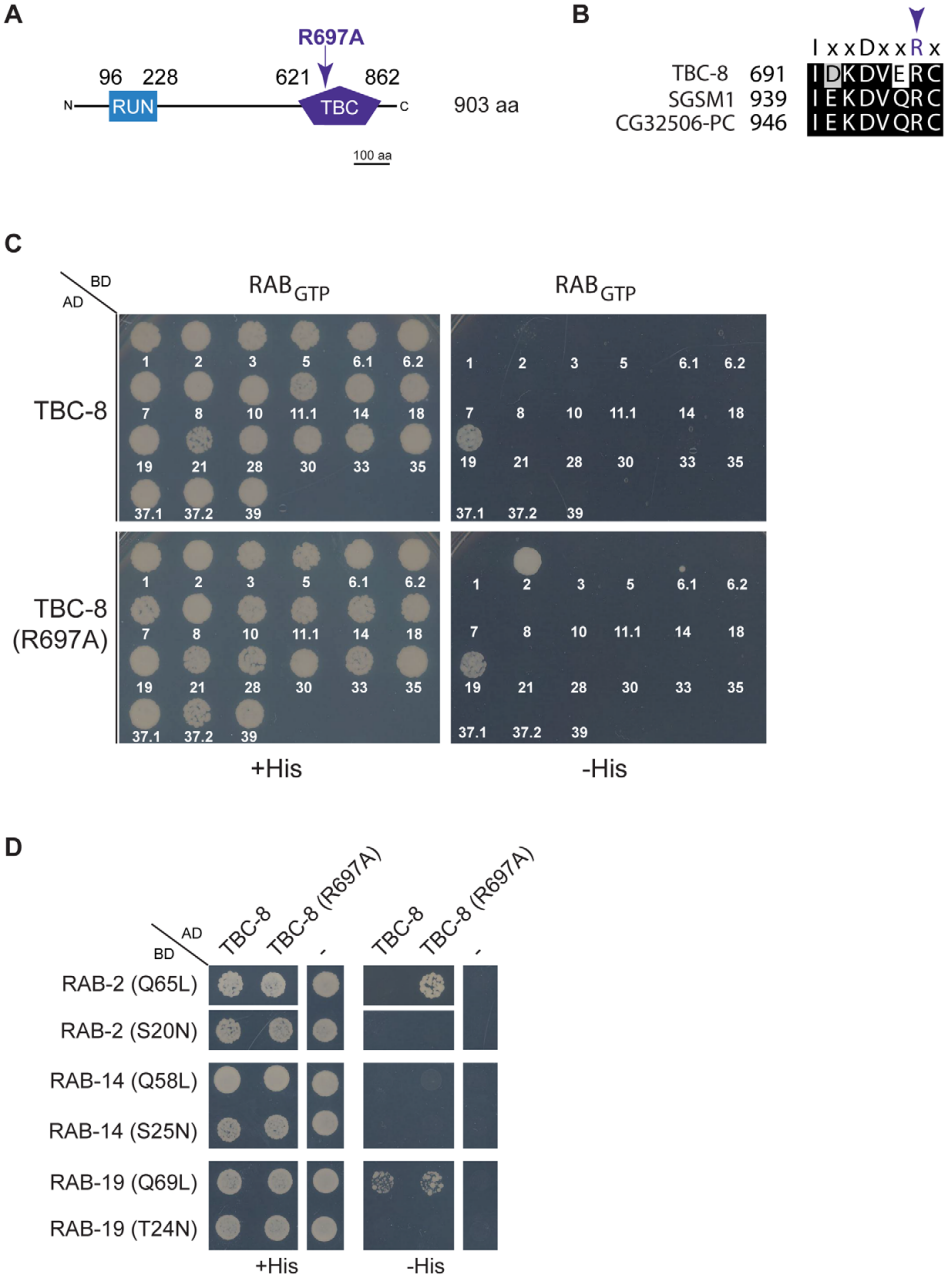
TBC-8 is a putative RAB-2 GAP. (A) TBC-8 contains two predicted domains: an N-terminal RUN domain (96 to 230 aa) (blue) and a C-terminal TBC-domain (621 to 862 aa) (purple). Mutation of the catalytically active arginine (R697) to alanine within the TBC-domain is indicated. Please see Figure S4 for full protein sequence. (B) Alignment of the catalytic motif of TBC-8 and its orthologs in humans (SGSM1) and in *Drosophila melanogaster* (CG32506-PC) are shown. The arrow indicates the catalytic arginine residue necessary for GAP activity. (C) In a yeast two-hybrid assay, all *C. elegans* Rabs in their constitutively GTP-bound form were tested against wild type TBC-8 (upper panel) and a catalytically inactive form of TBC-8 (R697A) (lower panel), respectively. Strikingly, RAB-2 (Q65L) interacted with TBC-8 (R697A) but not with wild type TBC-8, suggesting that TBC-8 is the GAP for RAB-2. Unlike RAB-2, RAB-19 (Q69L) interacted weakly with both forms of TBC-8. (D) Interactions of RAB-2 and RAB-19 with TBC-8 occurred in a GTP-dependent manner. Constitutively active RAB-2 (Q65L) and RAB-19 (Q69L) interacted with TBC-8 whereas their dominant inactive forms [RAB-2 (S20N), RAB-19 (T24N)] did not. The closest paralog of RAB-2, RAB-14, did not show interaction with TBC-8 wild type or R697A in a yeast two-hybrid analysis. AD: Gal4p DNA activation domain fusion, BD: Gal4p DNA binding domain fusion, His: histidine, RAB_GTP_: constitutively GTP-bound RAB GTPase, “–”: empty vector pGADT7 was used for testing self-activation.

It was previously shown that TBC domain-containing Rab GAPs possess an essential arginine finger located within the TBC-domain, which is crucial for its catalytic GAP activity [29] (Figure 6A). When mutated to alanine, the TBC-domain could still bind to its Rab partner but was unable to activate the intrinsic GAP activity of the Rab, which would lead to GTP hydrolysis [29]. TBC-8 also contains a conserved arginine finger residue (R697) (Figure 6A) within the catalytic motif that is conserved among its orthologs in humans (SGSM1) and *D. melanogaster* (CG32506-PC) (Figure 6B). If TBC-8 is the GAP specific for RAB-2, the interaction of both proteins should be detectable in the yeast two-hybrid system (Y2H) if the arginine finger of TBC-8 is mutated [29]. In order to identify the Rab/GAP pair, we screened TBC-8 and the catalytically inactive TBC-8 (R697A) with all constitutively active, GTP bound *C. elegans* Rab proteins in a Y2H experiment. Interaction of TBC-8 with Rab proteins was captured by growth on histidine selection plates. As expected, RAB-2 (Q65L) interacted with the catalytically inactive TBC-8 (R697A) but not with wild type TBC-8 (Figure 6C, 6D). This suggests that TBC-8 is a GAP for RAB-2 and the interaction between TBC-8 and RAB-2 is confined to the TBC-domain. In this screen, we also detected a specific binding of the GTP bound form of RAB-19 (Q69L) to TBC-8. However, in this case RAB-19 binds to both wild type TBC-8 and the catalytically inactive TBC-8 (R697A) (Figure 6C, 6D). This indicates that TBC-8 might be an effector and not a GAP of RAB-19. Furthermore, we could not detect NLP-21-derived VENUS trafficking defects in *rab-19* deletion mutants (Figure S7) indicating that TBC-8 together with RAB-19 has another role besides DCV trafficking. The co-localization and interaction studies strongly suggest that TBC-8 is the GAP for RAB-2 in motoneurons. However, we were unable to test the GAP activity biochemically since full-length TBC-8 as well as TBC-domain fragments were insoluble when purified from bacteria or insect cells.

### TBC-8 interacts with the RAB-2 effector RIC-19/ICA69

**Figure 7:**
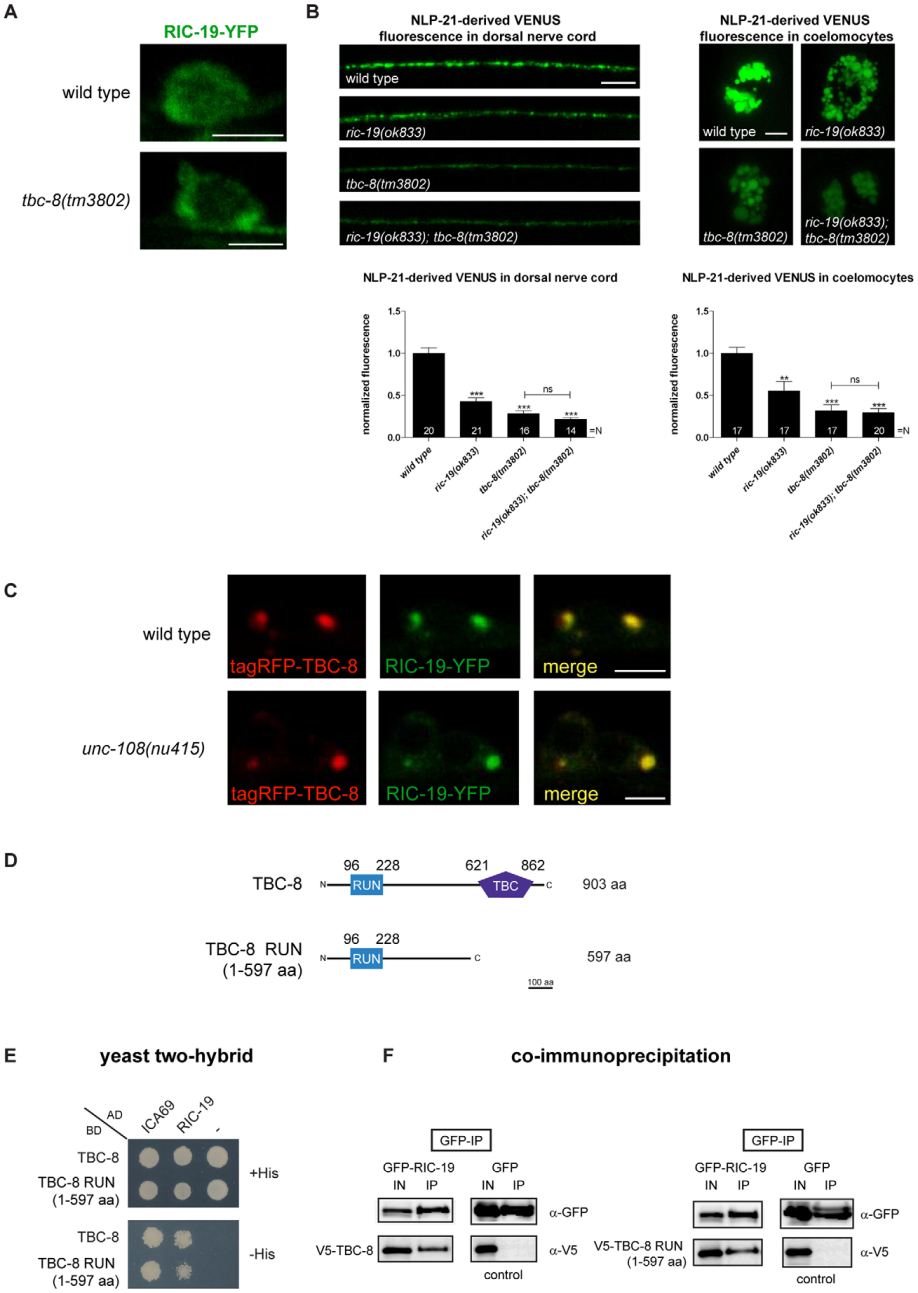
The RAB-2 effector, RIC-19/ICA69, interacts with TBC-8. (A) RIC-19-YFP is recruited to membranes in *tbc-8(tm3802)* mutants whereas it remained predominantly cytosolic in wild type neurons. Scale bar represents 3 µm. (B) NLP-21-derived VENUS analysis of the single mutants *ric-19(ok833), tbc-8(tm3802)* and the double mutant of both revealed that RIC-19 and TBC-8 are involved in the same genetic pathway. Scale bar represents 5 µm. Error bars = s.e.m. (***, P<0.0001; **, P<0.001, Student's t-test) (C) tagRFP-TBC-8 is able to recruit RIC-19-YFP to membranes resulting in a full co-localization in neurons. Scale bar represents 3 µm. This recruitment is also seen in an *unc-108/rab-2(nu415)* null mutant background. (D) Schematic representation of TBC-8 constructs (full-length and RUN domain (1–597 aa)) used for yeast two-hybrid analysis (Y2H) (E) and co-immunoprecipitation (co-IP) (F). (E) Y2H: RIC-19 and its human ortholog, ICA69, interacted with full length TBC-8 and TBC-8 RUN domain (1–597 aa) suggesting conservation of this interaction. (F) Co-IP: HEK293 cells were co-transfected with constructs expressing GFP-tagged RIC-19 (or GFP alone as control) and V5-TBC-8 (full length or RUN domain (1–597 aa)). An anti-GFP antibody was used to precipitate GFP-RIC-19 or GFP as control. Interactions between TBC-8 (full length and the RUN domain) with RIC-19 were visualized on Western blots. AD: Gal4p DNA activation domain fusion, BD: Gal4p DNA binding domain fusion, His: histidine, IN: Input, IP: immunoprecipitation, “– ”: empty vector pGADT7 was used for testing self-activation.

Previously, it has been shown that activated, GTP-bound RAB-2 recruits the BAR-domain (Bin/amphiphysin/Rvs) containing effector, RIC-19/ICA69, to Golgi membranes [22], [40]. We specifically showed that in the dominant active *unc-108/rab-2(n501)* allele, more RIC-19-YFP was membrane-bound [22]. A similar phenotype would be expected in *tbc-8* mutants where the RAB-2 GAP is missing. Thus, in *tbc-8* mutants RAB-2 should be mostly GTP-bound. As shown in Figure 7A, in *tbc-8(tm3802)* deletion mutants, RIC-19-YFP was indeed more punctate as compared to wild type. The extent of RIC-19-YFP membrane recruitment in *tbc-8* mutants is comparable to the dominant active allele of *unc-108(n501)* [22], [40]

To test genetically whether TBC-8 and RIC-19 are in the same pathway, we constructed a *tbc-8(tm3802); ric-19(ok833)* double mutant, which displayed the same DCV defects as *tbc-8* single mutants (Figure 7B). NLP-21-derived VENUS fluorescence was decreased in *tbc-8* mutants by 71.61±3.53%, in *tbc-8(tm3802); ric-19(ok833)* by 78.25±1.90%, whereas *ric-19(ok833)* single mutants showed a weaker decrease in fluorescence by 57.44±4.55%. These results suggest that TBC-8 and RIC-19 act in the same pathway.

We reasoned that if inactivation of TBC-8 leads to a constitutive activation of RAB-2 as indicated by RIC-19 recruitment, then overexpression of TBC-8 GAP should cause RIC-19 to be completely cytosolic. Unexpectedly, when tagRFP-TBC-8 was co-expressed with RIC-19-YFP, both proteins co-localized into huge cytosolic puncta in the cell bodies of motoneurons (Figure 7C, upper panel). This result suggested that TBC-8 and RIC-19 might form a complex that localizes to intracellular membranes. The extent of co-localization and the size of the puncta indicate that TBC-8 and RIC-19 strongly drive the membrane localization of the other. Interestingly, this cooperative membrane localization of TBC-8 and RIC-19 forced by overexpression is independent of RAB-2, since it is also seen in *unc-108/rab-2(nu415)* null mutant backgrounds (Figure 7C, lower panel). This result strongly suggested that RIC-19 and TBC-8 might directly interact. We tested this hypothesis by Y2H analysis as well as by co-immunoprecipitation (co-IP) of RIC-19 and TBC-8 in HEK293 cells (Figure 7D–7E). Both experiments revealed that there is a direct interaction between the RAB-2 effector, RIC-19, and the putative RAB-2 GAP, TBC-8. By Y2H analysis and co-IP, we showed that a fragment of TBC-8 (1–597 aa) containing the RUN domain was sufficient to bind to RIC-19 as well as to the human RIC-19 ortholog, ICA69 (Figure 7D–7E). Thus, the interaction between TBC-8 and RIC-19 seems to be evolutionarily conserved. Any attempt to shorten the TBC-8 RUN domain construct resulted in a loss of the interaction (data not shown). Therefore, our data suggest that active GTP-bound RAB-2 might recruit its own GAP via its effector RIC-19. However, this hypothesis has to be confirmed in further studies. It is also possible that another RAB-2 effector could also recruit TBC-8 in the absence of RIC-19, explaining the less severe DCV phenotype of *ric-19(ok833)* mutants.

## Discussion

### TBC-8 is a putative RAB-2 GAP specific to neurons

Previously, we have shown that RAB-2 is involved in neuronal DCV maturation in *C. elegans* and that a functional RAB-2 cycle is important to retain specific cargo in maturing DCVs [21], [22]. To gain insights into the spatio-temporal regulation of RAB-2 during DCV maturation, we screened all TBC domain-containing RAB GAPs in *C. elegans* for their involvement in DCV maturation. In this screen, we identified TBC-8, an evolutionarily conserved RAB GAP. We demonstrated that TBC-8 is an active RAB GAP since mutations in its catalytic motif “IxxDxxR” prevent the function of TBC-8 for DCV maturation. It was previously shown that mutation of the arginine within the catalytic motif to alanine renders the GAP into a catalytically inactive protein [29], [41]. TBC-8 was specifically expressed in neurons using a transcriptional reporter construct. The strong neuronal expression plus the similarity in DCV maturation defects suggested that TBC-8 is a neuron specific RAB-2 GAP. Furthermore, analysis of TBC-8 activity in non-neuronal tissues revealed that it does not regulate RAB-2 activity in postendocytic trafficking and in the removal of apoptotic cell corpses in the germ line. These results indicate that in non-neuronal tissues other GAPs exist that regulate RAB-2 activity. In addition, we cannot exclude that in different subtypes of neurons, RAB-2 function may be regulated by GAPs other than TBC-8.

Unfortunately, due to the inability of obtaining soluble TBC-8 when expressed in bacteria or insect cells, we were unable to demonstrate GAP activity towards RAB-2 *in vitro*. Thus, it is still possible that TBC-8 might not be a RAB-2 specific GAP.

However, three findings indicate that TBC-8 acts as a RAB-2 specific GAP *in vivo*.

First, the analysis of the two double mutants of *tbc-8* and *unc-108/rab-2* revealed that both proteins are involved in the same pathway. Second, in the yeast two-hybrid system, the GTP-bound form of RAB-2 specifically interacts with the TBC-domain of TBC-8 but only if the TBC-domain was rendered catalytically inactive. Previously, it was shown that an exchange of the catalytic arginine to alanine within the TBC-domain could be used to detect interaction of a GAP with its cognate Rab [41]. RAB-19 was observed to interact with both wild type and catalytically inactive forms of TBC-8 suggesting that it is unlikely that TBC-8 is the GAP for RAB-19. Third, TBC-8 influences the membrane localization of its binding partner RAB-2 when over-expressed in neurons. It was shown that active GTP-bound RAB-2 localizes to discrete puncta at the Golgi apparatus while inactive GDP-bound RAB-2 is mainly cytosolic [22]. However, when TBC-8 was over-expressed, RAB-2 was redistributed to the cytosol. Such redistribution of a Rab upon expression of its GAP has been previously used as an indication to screen for functional Rab/GAP pairs [42]. All these results strongly suggest that TBC-8 is likely to be a neuronal RAB-2 GAP. Irrespective of its specificity, the catalytic activity of the TBC-domain of TBC-8 is absolutely required for proper DCV maturation (Figure 1B, 1C). This strongly suggests that TBC-8 functions as an active Rab GAP during RAB-2 dependent DCV maturation in *C. elegans* motoneurons.

TBC-8 is conserved throughout evolution. There are two orthologs in mammals: SGSM1 (small G protein signaling modulator 1) also called RUTBC2 (RUN and TBC1 domain containing 2) [36] and SGSM2/RUTBC1 [36], [43]. Although TBC-8 shares domain structure with SGSM1 and SGSM2, it is more similar to SGSM1 based on sequence alignments. The TBC-domain of SGSM2 is about 162 aa longer than the TBC-domain of TBC-8, leading to a higher gap penalty when both proteins were aligned (data not shown). However, multiple splice variants can be found for both SGSM1 and SGSM2. Thus, we cannot exclude the possibility that one of those variants is even more closely related to TBC-8. SGSM1 is predominantly expressed in the adult brain [36]. Furthermore, it has been postulated to be involved in intracellular transport in neurons, where it localizes to the TGN [36]. It was shown that SGSM1 interacts with different Rab proteins; however, interactions with specific domains of SGSM1 were not shown [36]. In addition, SGSM1 binds to all members of the Rap GTPase family [36]. Recently, SGSM2/RUTBC1 has been found as a Rab9 effector [43]. Furthermore, functional GAP activity of SGSM2 towards Rab32 and Rab33 has been demonstrated *in vitro* [43]. The *C. elegans* genome does not encode a Rab9 ortholog, nor could we detect any interaction between TBC-8 and RAB-33 or GLO-1, the *C. elegans* Rab32 ortholog. Based on the fact that *Drosophila* contains two SGSM1/2 orthologs, it is likely that *C. elegans* has lost the SGSM2 ortholog along with Rab9 during evolution. Thus, we reason that SGSM1 is more closely related to TBC-8, consistent with their strong neuronal expression.

TBC-8 and its orthologs SGSM1/2 also contain an N-terminal RUN (RPIP8/UNC-14/NESCA) domain, which consists of α-helices [35], [44]. It is proposed that RUN domains are required to facilitate protein-protein interactions, specifically with small GTPases of the Rab and Rap family [34], [36], [45]. A fragment containing the RUN domain of SGSM1 was shown to interact with both Rap1 and Rap2 in a co-IP experiment [36]. We tested all *C. elegans* Rabs with TBC-8 and showed that RAB-19 binds to TBC-8, whereas no members of the *C. elegans* RAP family interacted with TBC-8 within the yeast two-hybrid system, suggesting that the interaction between the RUN domain and Rap GTPases might not be conserved (Figure S8).

### TBC-8 regulates DCV maturation

In this study, we demonstrated that *tbc-8(tm3802)* mutants have similar defects in DCV maturation as *unc-108/rab-2* mutants. Neuropeptides other than NLP-21 are also affected by *tbc-8* deletion. The fluorescence of VENUS-derived FMRF-like neuropeptide FLP-3 containing vesicles at the dorsal nerve cord was also decreased in *tbc-8* mutants. However, the fluorescence derived from the insulin-like neuropeptide, INS-22-VENUS, was unchanged in *tbc-8(tm3802)*, similar to *unc-108/rab-2* [21]. Unlike NLP-21 and FLP-3, the insulin-like neuropeptide INS-22 lacks PC2 (proprotein convertase 2) cleavage sites. Therefore, VENUS is not cleaved off during the maturation process, and the VENUS-tag is aggregated together with neuropeptides within the insoluble core of DCVs [31], [46]. These data further suggest that it is mainly the soluble cargo that is lost in *tbc-8* mutants, as observed also in the *unc-108/rab-2* mutants.

Here, we could not observe any differences in the axonal fluorescence levels of the DCV integral membrane protein, IDA-1-GFP, indicating that transmembrane cargos are not affected by *tbc-8* deletion. However, in *unc-108/rab-2* mutants, the axonal fluorescence of the transmembrane cargo IDA-1-GFP is decreased [21]. This result suggests that both soluble and transmembrane cargos are possibly lost from DCVs in *unc-108/rab-2* mutants [21]. This discrepancy in transmembrane cargos being affected in *unc-108* mutants but not in *tbc-8* mutants suggests that in neurons, another GAP may exist at the site where RAB-2 functions to retain transmembrane cargos. Despite this difference, the axonal DCV numbers are not affected by *rab-2/unc-108* or *tbc-8* mutations as determined by EM. This finding demonstrates that TBC-8 participates with RAB-2 in the retention of specific (soluble) cargo in DCVs during the maturation process. Since a blockade of the endosomal delivery by over-expression of constitutively active GTP-bound RAB-5 (Q78L) could rescue the loss of soluble NLP-21-derived VENUS, TBC-8 and RAB-2 are likely involved in cargo sorting processes during maturation at the Golgi-endosomal interface.

Collectively, our results indicate that *tbc-8(tm3802)* and *unc-108/rab-2(n501)* dominant active mutants share similar defects in DCV maturation, reiterating the idea that TBC-8 is the GAP for RAB-2. Despite the similarities in DCV maturation phenotypes, there are major differences in behavioral phenotypes in both *tbc-8(tm3802)* and *unc-108/rab-2(n501)* mutants. Unlike *unc-108/rab-2* mutants, *tbc-8(tm3802)* mutants display no defects in locomotion behavior (Figure S3) [21], [22]. There are several possible explanations why both mutants show different locomotion phenotypes. First, *tbc-8* may not be expressed in all subtypes of neurons that are necessary to coordinate locomotion, whereas *unc-108/rab-2* is more broadly expressed in (all) neurons and other tissues [22], [33]. Interestingly, it was shown that the locomotory defect in *unc-108/rab-2* mutants could be rescued by expressing *unc-108* under the pan-neuronal synaptobrevin-1 (*snb-1*) promoter, but not when *unc-108* was expressed under the *unc-17* promoter, which is expressed in a subtype of motoneurons, the cholinergic motoneurons [33]. Secondly, in the absence of *tbc-8* in some neurons, another GAP might substitute for TBC-8. This hypothesis is further supported by the fact that the transmembrane protein IDA-1 is unaffected by *tbc-8* deletion, suggesting that another GAP must exist in neurons. Thirdly, in the absence of TBC-8, it is possible that the low intrinsic activity of RAB-2 might partly rescue some of the phenotypes in *tbc-8* mutants that are observed in *unc-108/rab-2* mutants. Fourthly, an unknown transmembrane factor, which is normally present on DCVs could be lost in *unc-108/rab-2* mutants during DCV maturation and thus be responsible for the uncoordinated phenotype. This transmembrane factor could still be present in *tbc-8* mutants since no defects in IDA-1 trafficking were observed in *tbc-8* mutants.

### The RIC-19-TBC-8 RAB-2 effector complex is required for proper DCV maturation

How does the possible RAB-2 GAP, TBC-8, regulate RAB-2 activity during DCV maturation? We have previously shown that RAB-2 dependent retention is achieved by a dynamic ON/OFF cycle of RAB-2 at the Golgi-endosomal interface [21], [22]. Thus, RAB-2 most likely goes through several rounds of activation and deactivation during DCV maturation. The fact that the RAB-2 effector, RIC-19, interacts with the RAB-2 GAP, TBC-8, supports this view. Thus, once RAB-2 is locally activated at the Golgi-endosomal interface, it recruits the RIC-19/TBC-8 complex, which in turn will lead to its deactivation and release from its membrane localization. This will also then lead to a cytosolic redistribution of RIC-19 and TBC-8. The fact that overexpressed RIC-19 and TBC-8 show a dramatic membrane localization even in the absence of RAB-2 suggests that RIC-19 and TBC-8 might also bind to a yet unidentified factor that is membrane-localized, independent of RAB-2. The observation that this massive membrane localization of RIC-19 and TBC-8 is only seen by overexpression of both proteins suggests that the interactions might be of low affinity. Thus, in a normal situation, the effective concentrations would be insufficient for a productive interaction and thus recruitment. In this case, the interaction would require activated RAB-2 for efficient recruitment of RIC-19 and TBC-8 to membranes. This would postulate that active GTP-bound RAB-2 would interact with this unknown factor as well as with the RIC-19/TBC-8 complex. Accordingly, an inactivation of RAB-2 by its GAP, TBC-8, would lead to a rapid disassembly of the complex and a release of its components for a new round of recruitment. However, from our current set of data, we cannot fully exclude the possibility that RIC-19 and TBC-8 are recruited separately from each other to the Golgi-endosomal interface and start to interact at RAB-2 positive vesicles. More efforts have to be made to solve the function of the TBC-8/RIC-19 interaction.

It is currently unclear whether and how TBC-8 GAP activity is regulated. On the one hand, TBC-8 can be inactive when recruited by active RAB-2 via RIC-19 and then activated after recruitment. On the other hand, TBC-8 can be constitutively active while bound to RIC-19, which then would lead to an immediate deactivation of RAB-2 after recruitment of the effector complex. RIC-19 binds outside of the TBC-domain, to the N-terminal part of TBC-8 containing the RUN domain. Thus, it is currently unlikely that RIC-19 would mask the TBC-domain and inhibit GAP activity. The interaction between RIC-19 and TBC-8 seems to be conserved because the human homolog of RIC-19, the diabetes autoantigen ICA69, could also bind to TBC-8 in the yeast two-hybrid system. This result suggests that TBC-8 and its interaction to ICA69 may also play a role in insulin signaling in humans.

TBC-8 activity might also be regulated by its interaction with active GTP-bound RAB-19. However, we did not detect any defects in DCV maturation in *rab-19(ok1845)* deletion mutant animals. This result suggests that RAB-19 is not involved in DCV trafficking and that TBC-8 together with RAB-19 might act in another DCV-independent pathway.

So far it has been described that in Rab GAP cascades, the downstream Rab would recruit the Rab GAP for the upstream Rab via its effector complex [25]. For example, in the Ypt1p/Ypt32p cascade Ypt1p associates with the Golgi and regulates ER to Golgi trafficking [47]. The GAP for Ypt1, Gyp1p, is recruited by interaction with activated Ypt32p, the downstream Rab [48]. It has been shown that this counter-current GAP cascade limits the overlap between activated Ypt1p and Ypt32p within the yeast secretory pathway [49], [50]. Therefore, this GAP cascade sharpens the boundaries during the Rab conversion cascades supporting sorting of cargo into the downstream Rab domain and thus, enhancing the directionality of the transport process [51].

However, the situation might be different in the case of RAB-2 and TBC-8 where the active Rab GTPase would recruit its own GAP through its effector complex. This would suggest that RAB-2 might not participate in a traditional Rab conversion cascade. Further support for this view comes from the fact that we were unable to identify a second Rab GTPase in *C. elegans* showing the same DCV maturation phenotype despite screening all Rab GTPases in *C. elegans* (our unpublished data). It is therefore likely that RAB-2 participates in a membrane trafficking mechanism distinct from a Rab cascade. However, since we were unable to test RAB-2 specific GTPase activity of TBC-8 due to protein insolubility, we can currently not exclude that RAB-2 might be part of a Rab cascade. RAB-2 might participate in a highly dynamic membrane sorting event by helping to transiently orchestrate the assembly of an acceptor complex required for DCV maturation. This complex would then subsequently be rapidly disassembled to allow sorting to proceed further. It is conceivable that active RAB-2 would help to accept retrograde trafficking carriers within the Golgi-endosomal interface to be delivered back to the maturing DCV compartment. This would explain why in *rab-2* mutants, cargo from maturing DCV is lost to the endosomal-lysosomal pathway [21], [22].

The fact that TBC-8 also binds to human ICA69 might suggest that the role of TBC-8 is evolutionarily conserved and that its human ortholog may play an important role in regulating insulin secretion from pancreatic ß-cells since SGSM1 is also expressed in the pancreas [36].

## Materials and Methods

### Strain, crosses, injections

*C. elegans* strains were maintained at 20°C on nematode growth medium (NGM) seeded with *Escherichia coli* OP50 as described previously [52]. The following strains were used in this study: wild type *N2 Bristol* strain, *tbc-8(tm3802)*, *unc-108(n501)*, *unc-108(nu415)*, *egl-3(gk238)*, *ric-19(ok833)*, *rab-19(ok1845)*, *eri-1(mg366)*, *tbc-1(tm2282)*, *tbc-2(qx20)*, *tbc-4(tm3255)*, *tbc-11(ok2576)*, *tbc-12(gk362)*, *tbc-13(ok1812)* and *tbc-18(ok2374)*. The following integrated transgenic lines were used in this study: *nuIs183[punc-129::nlp-21–venus]*, *nuIs195[punc-129::ins-22–venus]*, *ceIs61[punc-129::flp-3–venus]*, *ceIs72[punc-129::ida-1–gfp]*, *nuIs152[punc-129::gfp-snb-1]*, *nuIs168[punc-129::venus-rab-3]*, *bIs34[*p*rme-8::rme-8-gfp]*, *arIs36[*p*hsp::ssgfp]*, *arIs37[*p*myo-3::ssgfp]* and *bcIs39[*p*lim-7::ced-1-gfp]*. All these integrated strains were crossed into the strain *tbc-8(tm3802)*. Crosses were carried out using classical genetic approaches, and the progeny was genotyped by PCR. For more detailed information about strains used see Table S1. Transgenic worm lines were generated by microinjections into *N2 Bristol* strain or into *tbc-8(tm3802)* [53]. Table S2 lists all transgenic worm lines including the plasmids and co-injection marker concentrations used in this study.

### Molecular cloning

The cDNA of *tbc-8* was amplified by PCR using a cDNA library (ProQuest, Invitrogen) with the primers oGQ1622 (tt ccc ggg tta ccg gtt atg tgg agg gcg aag aag cca aca) and oGQ1623 (ggg gcg gcc gcc ctc gag cta ctt gag gtg ttg cac aag gtt) and TA cloned into the vector pGEMT (Promega). Positive clones were verified by sequencing reaction (Qiagen). *tbc-8* was then subcloned into different vectors: see Table S3 for details. For expression of genes of interest in the nervous system of *C. elegans*, the backbone of the vector *pPD115.62* was used. The *myo-3* promoter in *pPD115.62* was exchanged for the *rab-3* promoter using *Pst*I and *Kpn*I restriction sites creating *prab-3::gfp*. The same procedure was used to generate *punc-129::gfp*. For the *tbc-8* promoter construct, a fragment of 2873 bp upstream of *tbc-8* start codon together with the first five *tbc-8* codons was PCR amplified using N2 genomic DNA with the following primer pair: oGQ1793 (ctt aag ctt ctg cag gaa ctt ttc cat ctg) and oGQ2055 (agt aac cgg tgc cct cca cat atc tgc cga tga atg ccg).

The catalytic arginine finger of TBC-8 was mutated to alanine using a site-directed mutagenesis approach. The following primers were used: oGQ1622, oGQ1623, oGQ1698 (gac gtg gag gca tgc gat aga aat ttg atg ttc) and oGQ1699 (tct atc gca tgc ctc cac gtc ctt gtc aat tct).

For yeast two-hybrid analysis, all dominant active forms of *rab* genes were cloned into the bait vector pGBKT7 whereas all *tbc-8* variants were cloned into the prey vector pGADT7 (Clontech) (Table S3).

### Fluorescence imaging and quantitative analysis

For confocal microscopy, live worms were paralyzed with 50 mM NaN_3_ (Sigma) on 2% agarose (Invitrogen) pads. An inverted Confocal Laser Scanning Microscope (SP2, Leica) with a 100× oil objective (NA = 1.4) (co-localization studies) or a 63× oil objective (NA = 1.32) (DCV assay, expression pattern studies) was used. GFP was excited with a laser at 488 nm, YFP at 514 nm and tagRFP as well as mCherry at 561 nm. The scan was performed with a resolution of 1024×1024 pixels, and the pinhole was set to 1 airy unit. To study co-localization of TBC-8 with different subcellular markers tagged with fluorescent proteins, images of neuronal cell bodies from the ventral nerve cord were taken. Image stacks were captured and average intensity projections were obtained using the Leica software. These images were then edited using ImageJ software (National Institutes of Health).

Furthermore, images of expression pattern of *tbc-8* were taken using a Perkin Elmer Spinning Disc Confocal Microscope. These images were edited using Adobe Photoshop software.

For quantification studies from DNC, neuronal cell bodies and coelomocytes (DCV assay, imaging of endocytosed ssGFP in coelomocytes secreted from muscle cells), young adult worms were imaged as described previously [31]. For cell body and DNC imaging, the neuronal cell bodies and DNC were oriented toward the objective, whereas for coelomocytes imaging, the posterior coelomocytes were oriented laterally. Image stacks of the regions of interest were captured and maximum intensity projections were obtained using the Leica software. For all obtained images, the same settings were used. These projections were thresholded and quantified using the ImageJ software (National Institutes of Health). All these data were normalized to wild type.

For quantification of vesicle sizes in neuronal cell bodies in *nuIs183* background strains, obtained images of cell bodies (explained above) were analyzed using the ImageJ software (National Institutes of Health).

In order to image apoptotic cell corpses, image stacks of the distal gonad arms were captured and projections were obtained using the Perkin Elmer Spinning Disc Confocal Microscope. From these pictures the number of cell corpses in the distal gonad arm and in the gonad loop was counted manually for each strain.

### Yeast two-hybrid

The Matchmaker yeast two-hybrid assay was performed according to the manufacturer's protocol (Clontech). The appropriate plasmid combinations were transformed into the yeast strain *AH109* (Clontech) and spread onto selective growth media lacking leucine and tryptophan for plasmid selection. Protein interactions were tested as follows: several clones of transformants were mixed and diluted to OD_600_ of 0.4 (RAB interaction studies) or 0.2 (RAP interaction studies). Five microliters of this yeast dilution was spotted onto selective plates lacking leucine, tryptophan and histidine. Interactions were identified by growth after 3–4 days. All interacting proteins were tested for self-activation as described above using the appropriate empty vector pGBKT7 or pGADT7, respectively.

### Co-immunoprecipitation and immunoblotting

HEK293 cells were grown in high glucose (4.5 g/l) DMEM supplemented with 10% FBS, 110 mg/l sodium pyruvate, 2 mM glutamine, 100 U/ml penicillin, and 10 µg/ml streptomycin in a 5% CO_2_ incubator at 37°C.

For co-immunoprecipitation, 4×10^6^ HEK293 cells were plated onto two 10 cm petri dishes 24 hours before transfection, which was performed using TurboFect *in vitro* Transfection Reagent according to the manufacturer's protocol (Fermentas). After 24 hours, cells were washed with PBS and harvested in lysis buffer (50 mM Tris pH 7.5, 150 mM NaCl, 1% Triton X 100, 0.5 mM EDTA, 10% glycerol, Complete Mini Protease inhibitor (Roche)) for 30 min at 4°C. Lysates were pre-cleared by centrifugation at 4°C before supernatant was incubated with 2 µg monoclonal anti-GFP antibody (clone 3E6, Invitrogen) for 3 hours at 4°C. Protein G Plus-sepharose (Pierce) beads were added. After another incubation time of 2 hours, the beads were washed three times with washing buffer (50 mM Tris pH 7.5, 500 mM NaCl, 0.1% Triton X 100, 0.5 mM EDTA, 10% glycerol, Complete Mini Protease inhibitor (Roche)) and resuspended in Laemmli loading buffer. Samples were resolved on 10% SDS-polyacrylamide gels and blotted onto a nitrocellulose membrane. The detection of co-precipitated proteins were performed by applying a mixture of two monoclonal mouse anti-GFP antibody (1∶1000) (clones 7.1 and 13.1, Roche) and monoclonal anti-V5 antibody (1∶5000) (Invitrogen) followed by goat anti-mouse horseradish peroxidase-conjugated secondary antibody (1∶10,000) (Jacksons Laboratory). A FujiFilm LAS 3000 processor was used to develop images, which were edited using the ImageJ software (National Institutes of Health).

### High pressure freezing, freeze substitution, electron microscopy

A 100 µm deep aluminum platelet (Microscopy Services, Flintbek) was filled with *E. coli* OP50 suspension. About 20 young adult worms were transferred into the chamber and immediately frozen using a BalTec HPM 10. Freeze substitution was carried out in a Leica AFS2. Incubations were performed at −90°C for 100 h in 0.1% tannic acid, 7 h in 2% OsO_4_, and at −20°C for 16 h in 2% OsO_4_, followed by embedding in EPON at room temperature [54] (all solutions w/v in dry acetone). Fifty nanometer sections were mounted on copper slot grids and placed for 10 min on drops of 4% (w/v) uranyl acetate in 75% methanol and then washed in distilled water. After air drying, the grids were placed on lead citrate [55] for 2 min in a CO_2_-free chamber, and rinsed in distilled water. Micrographs were taken with a 1024×1024 CCD detector (Proscan CCD HSS 512/1024; Proscan Electronic Systems, Scheuring, Germany) in a Zeiss EM 902A, operated in the bright field mode. The SV and DCV diameter and distribution at the synapse of motoneurons were analyzed by a semi-automated analysis software XtraCount (manuscript in preparation).

For the analysis of presynaptic terminals and SV and DCV distributions, cross sections of young adult animals were used to image cholinergic neuro-muscular junction (NMJ) synapses in the ventral nerve cord, posterior to the nerve ring. These cholinergic NMJ synapses were defined as polyadic synapses projecting onto muscle arms as well as other neurons according to the standard convention in the *C. elegans* EM field. Axons showing a clearly visible presynaptic density and synaptic vesicles were defined as synapse. The mean area of presynaptic terminal was measured in cross sections using the surrounding axonal membrane as border.

For the morphological analysis of neurons, ten motoneuronal cell bodies localized in the ventral nerve cord in wild type worms were compared with seven cell bodies in *tbc-8(tm3802)* worms.

### Texas red-conjugated BSA endocytosis assay

For the analysis of postendocytic trafficking within coelomocytes of the fluid-phase endocytosis marker TR-BSA, the integrated strain *bIs34[*p*rme-8::rme-8-GFP]* was crossed into *tbc-8(tm3802)* to label RME-8 positive endosomes. TR-BSA (1 mg/ml) was injected into the body cavity in the pharyngeal region of young adult worms as described previously [56]. Uptake and postendocytosis was analyzed after 10, 30 and 50 min after injection by confocal microscopy. At least five animals were injected for each time point. Single fluorescence images at the middle plane of each coelomocyte were taken and line-averaged. Images were edited using ImageJ software (National Institutes of Health).

### ssGFP endocytosis assay

The strain *arIs36[*p*hsp::ssGFP]* [39] was crossed into *tbc-8(tm3802)*. Young adult worms were grown at 20°C before a heat-shock at 33°C for 30 min was performed. Worms were put back to 20°C to recover until they were used for imaging. The uptake of ssGFP into coelomocytes and degradation of endocytosed GFP was monitored after 3.5, 6 and 28 hours after heat-shock. All fluorescence pictures were taken with the same settings. At the time points where fluorescence was hard to detect during imaging, DIC (differential interference contrast) images of the respective specimen were taken. Later, these images were used to outline the cell boundaries of coelomocytes. Images were edited using the ImageJ software (National Institutes of Health).

### RNAi by feeding

RNAi by feeding was performed as described previously [57]. The plasmids L4440 and L4440-*tbc-8* were transformed into the *E. coli* strain HT115, respectively. Overnight cultures of these bacteria were seeded onto NGM plates that contained 100 mg/ml ampicillin and 1 mM IPTG. Ten L4 worms of the strain *eri-1(mg366);nuIs183* were placed onto these plates to allow egg laying and were transferred onto new RNAi plates every 12 hours. After the third round of transferring worms, the parents were removed and the progeny was imaged when they reached the young adult worm stage. Fluorescence of NLP-21-derived VENUS in the dorsal nerve cord was normalized to the VENUS fluorescence of mock RNAi (L4440) worms.

### Movement assay

In order to assay locomotion, young adult worms of each strain were transferred to non-seeded NGM plates. After an initial adjustment time of 30 min, the number of body bends (one sine wave) was counted over a period of 3 min.

## Supporting Information

Figure S1 Analysis of mutants of TBC-domain containing GAPs in *C. elegans* for DCV trafficking defects of the NLP-21-VENUS marker. This assay revealed that only *tbc-8(tm3802)* deletion mutants displayed decreased fluorescence levels of VENUS derived from NLP-21 in the dorsal nerve cord similar to *unc-108/rab-2* mutants (Figure 1B). Error bars = s.e.m. (***, P<0.0001; ANOVA with Bonferroni post test).
(PDF)

Figure S2 RNAi of *tbc-8* leads to decreased fluorescence levels of VENUS derived from NLP-21 in the dorsal nerve cord. Similar results were observed in *tbc-8(tm3802)* deletion mutants (Figure 1B). Downregulation of *tbc-8* expression in an *eri-1(mg366); nuIs183* background caused a decreased VENUS fluorescence level in the dorsal nerve cord by 37.67±5.80% compared to the control strain. Control: The mock vector (L4440) was used. Scale bar represents 5 µm. Error bars = s.e.m. (***, P<0.0001; Student's t-test).
(PDF)

Figure S3 *tbc-8(tm3802)* mutants do not display movement defects. Young adult worms of each strain were transferred to non-seeded plates, adjusted for several minutes before the number of body bends per min of each worm was recorded. *tbc-8* mutants displayed normal rate of locomotion when compared to wild type animals. Error bars = s.e.m. (ns, P>0.05; Student's t-test).
(PDF)

Figure S4 Protein sequence alignment of TBC-8 with its orthologs SGSM1 (*H. sapiens*) and CG32506-PC (*D. melanogaster*). Multiple sequence alignment was performed using the program MUSCLE and displayed using the program BOXSHADE. The predicted domains are color-coded. The RUN domain is shown in blue and the conserved blocks forming the ‘core’ of the RUN domain (A–F) are highlighted in black lines [35]. The TBC-domain (prediction made by the SMART program) is depicted in purple. The catalytic arginine residue, R697, is marked by a yellow arrowhead within its catalytic motif (yellow line). Note: domain lengths predicted for TBC-8 are shown. Accession number of SGSM1: NP_001035037; FlyBase ID of CG32506-PC: FBpp0300194.
(PDF)

Figure S5 Postendocytic trafficking in *tbc-8* mutants is not affected. (A) *tbc-8(tm3802)* mutants were crossed into the strain *arIs37[*p*myo-3::ssGFP]* that constitutively expresses ssGFP from muscle cells. The fluorescence of endocytosed GFP in coelomocytes of *tbc-8* mutants was imaged and compared to levels of endocytosed GFP in a wild type background. Representative pictures of the steady-state endocytosis of ssGFP in coelomocytes are shown. Scale bar represents 5 µm. Error bars = s.e.m. (ns, P>0.05, Student's t-test) (B) The strain *arIs36[*p*hsp::ssGFP]* was crossed into *tbc-8(tm3802)*. After a short heat-shock, both strains were monitored for uptake of ssGFP into coelomocytes and degradation of endocytosed GFP after various time points (3.5 hours, 6 hours and 28 hours). All fluorescence pictures were taken with the same settings. Dashed lines indicate outlines of coelomocytes. Scale bar of worm sections represent 50 µm. Scale bar of coelomocytes represent 5 µm. (C) The fluid-phase endocytosis marker TR-BSA was injected into the body cavity of *tbc-8(tm3802)* worms and its fate within coelomocytes was followed over time (10 min, 30 min, 50 min). For this purpose, the strain *bIs34[*p*rme-8::rme-8-gfp]*, which labels RME-8 positive endosomes, was crossed into *tbc-8(tm3802)*. After 10 min, TR-BSA (red) was endocytosed and was visible in RME-8-GFP (green) positive vesicles in both *tbc-8(tm3802)* mutants and in wild type worms. Therefore, endocytosis of the fluid-phase marker seemed to be unaffected in *tbc-8(tm3802)* mutants. Further observation of the kinetics in postendocytic trafficking of TR-BSA (30 min, 50 min) did not revealed any defects in *tbc-8(tm3802)* mutants. Scale bar represents 5 µm.
(PDF)

Figure S6 *tbc-8(tm3802)* mutants do not show defects in degradation of apoptotic cell corpses in the germ line. CED-1-GFP (*bcIs39*; [58]) was used as a marker to visualize apoptotic cell corpses in the germ line of *C. elegans*. Imaging stacks of one gonad arm were captured and the number of CED-1-GFP positive apoptotic cell corpses was recorded. *tbc-8(tm3802)* mutants displayed similar numbers of apoptotic cell corpses like wild type worms, whereas *unc-108(nu415)* mutants have defects in the engulfment of apoptotic cell corpses resulting in high numbers of corpses in their gonad arms, which was described previously [37], [38]. Scale bar represents 20 µm. Error bars = s.e.m. (***, P<0.0001; Student's t-test).
(PDF)

Figure S7 *rab-19(ok1845)* mutants do not display defects in DCV trafficking of the NLP-21-VENUS marker in the dorsal nerve cord. *rab-19(ok1845)* mutants have similar fluorescence levels of NLP-21-derived VENUS (89.83±10.69%) in the dorsal nerve cord like wild type worms. Scale bar represents 5 µm. Error bars = s.e.m. (ns, P>0.05; Student's t-test).
(PDF)

Figure S8 TBC-8 does not interact with RAP proteins in a yeast two-hybrid analysis. All three *C. elegans* RAP proteins, RAS-1 and RAL-1 in their native (upper panel) and their predicted activated state (lower panel) [59], [60], [61] were tested for interaction with TBC-8(R697A) in a yeast two-hybrid analysis. No growth on histidine-lacking plates was observed after 3 to 4 days. AD: Gal4p DNA activation domain fusion, BD: Gal4p DNA binding domain fusion, His: histidine.
(PDF)

Table S1 Strains used in this study.
(PDF)

Table S2 Transgenic arrays used in this assay.
(PDF)

Table S3 Constructs used in this study.
(PDF)
