# Supplementary figures and images for: TBC-8, a Putative RAB-2 GAP, Regulates Dense Core Vesicle Maturation in Caenorhabditis elegans
Source: PLoS Genet. 2012 May 24;8(5):e1002722. doi: 10.1371/journal.pgen.1002722 (PMC3359978; doi:10.1371/journal.pgen.1002722)

NLP-21-VENUS in dorsal nerve cord

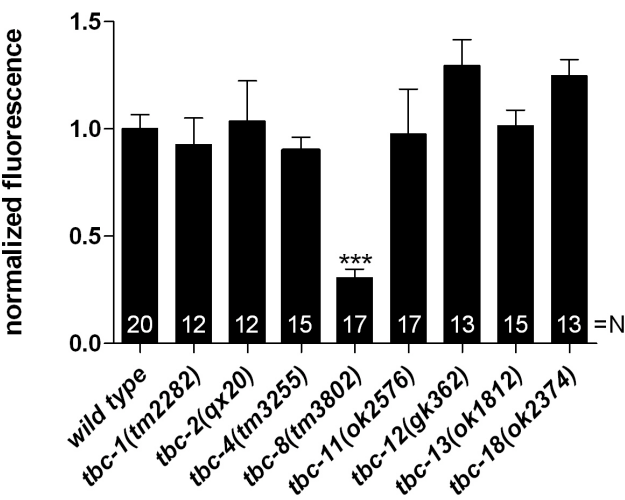

Supplement: Figure S1 — Analysis of mutants of TBC-domain containing GAPs in C. elegans for DCV trafficking defects of the NLP-21-VENUS marker. This assay revealed that only tbc-8(tm3802) deletion mutants displayed decreased fluorescence levels of VENUS derived from NLP-21 in the dorsal nerve cord similar to unc-108/rab-2 mutants (Figure 1B). Error bars = s.e.m. (***, P<0.0001; ANOVA with Bonferroni post test). (PDF) [file pgen.1002722.s001.pdf]

NLP-21-derived VENUS fluorescence in dorsal nerve cord

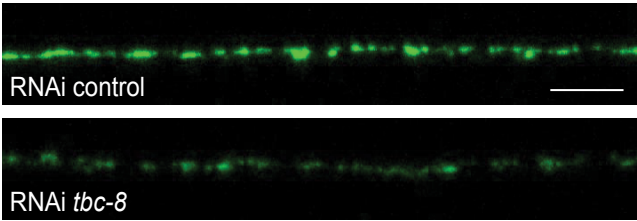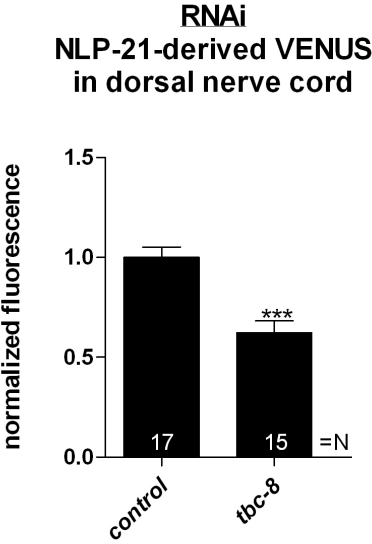

Supplement: Figure S2 — RNAi of tbc-8 leads to decreased fluorescence levels of VENUS derived from NLP-21 in the dorsal nerve cord. Similar results were observed in tbc-8(tm3802) deletion mutants (Figure 1B). Downregulation of tbc-8 expression in an eri-1(mg366); nuIs183 background caused a decreased VENUS fluorescence level in the dorsal nerve cord by 37.67±5.80% compared to the control strain. Control: The mock vector (L4440) was used. Scale bar represents 5 µm. Error bars = s.e.m. (***, P<0.0001; Student's t-test). (PDF) [file pgen.1002722.s002.pdf]

## Movement Assay

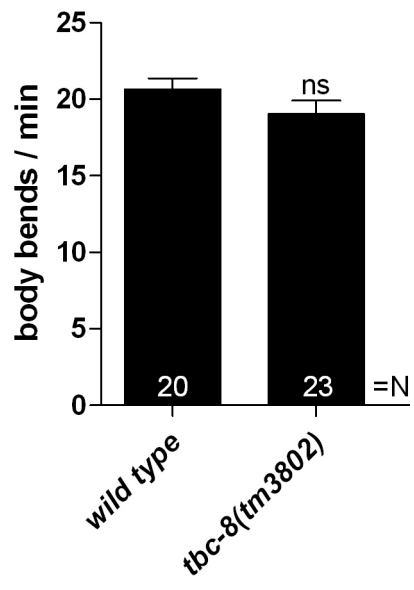

Supplement: Figure S3 — tbc-8(tm3802) mutants do not display movement defects. Young adult worms of each strain were transferred to non-seeded plates, adjusted for several minutes before the number of body bends per min of each worm was recorded. tbc-8 mutants displayed normal rate of locomotion when compared to wild type animals. Error bars = s.e.m. (ns, P>0.05; Student's t-test). (PDF) [file pgen.1002722.s003.pdf]

**A** endocytosed ssGFP in coelomocytes

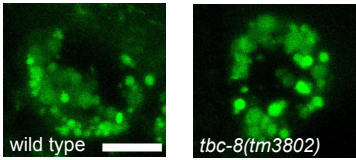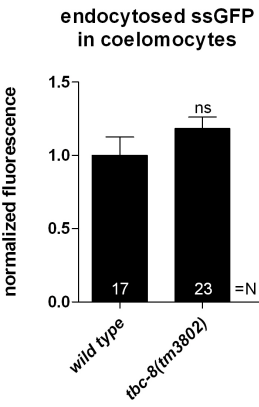

**B**

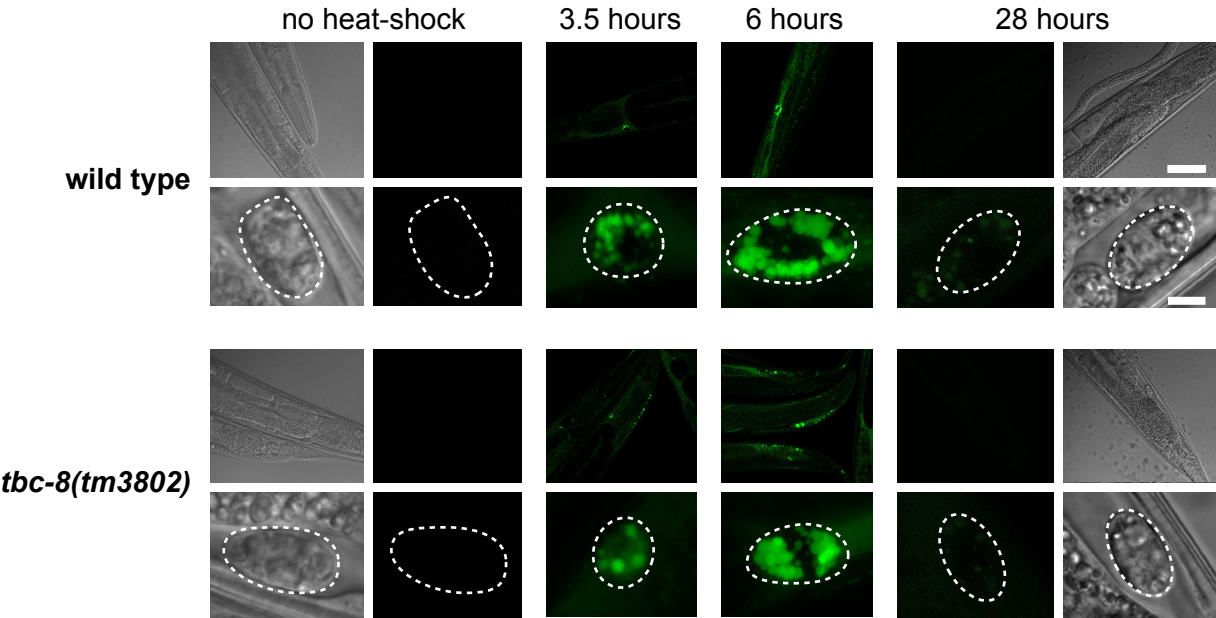

**C**

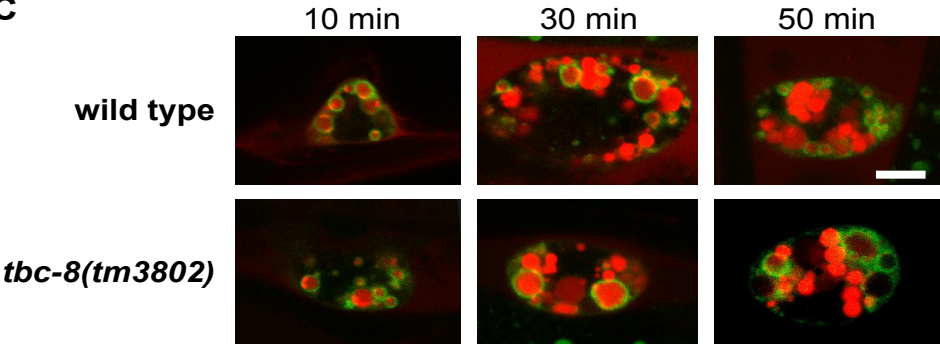

Supplement: Figure S5 — Postendocytic trafficking in tbc-8 mutants is not affected. (A) tbc-8(tm3802) mutants were crossed into the strain arIs37[pmyo-3::ssGFP] that constitutively expresses ssGFP from muscle cells. The fluorescence of endocytosed GFP in coelomocytes of tbc-8 mutants was imaged and compared to levels of endocytosed GFP in a wild type background. Representative pictures of the steady-state endocytosis of ssGFP in coelomocytes are shown. Scale bar represents 5 µm. Error bars = s.e.m. (ns, P>0.05, Student's t-test) (B) The strain arIs36[phsp::ssGFP] was crossed into tbc-8(tm3802). After a short heat-shock, both strains were monitored for uptake of ssGFP into coelomocytes and degradation of endocytosed GFP after various time points (3.5 hours, 6 hours and 28 hours). All fluorescence pictures were taken with the same settings. Dashed lines indicate outlines of coelomocytes. Scale bar of worm sections represent 50 µm. Scale bar of coelomocytes represent 5 µm. (C) The fluid-phase endocytosis marker TR-BSA was injected into the body cavity of tbc-8(tm3802) worms and its fate within coelomocytes was followed over time (10 min, 30 min, 50 min). For this purpose, the strain bIs34[prme-8::rme-8-gfp], which labels RME-8 positive endosomes, was crossed into tbc-8(tm3802). After 10 min, TR-BSA (red) was endocytosed and was visible in RME-8-GFP (green) positive vesicles in both tbc-8(tm3802) mutants and in wild type worms. Therefore, endocytosis of the fluid-phase marker seemed to be unaffected in tbc-8(tm3802) mutants. Further observation of the kinetics in postendocytic trafficking of TR-BSA (30 min, 50 min) did not revealed any defects in tbc-8(tm3802) mutants. Scale bar represents 5 µm. (PDF) [file pgen.1002722.s005.pdf]

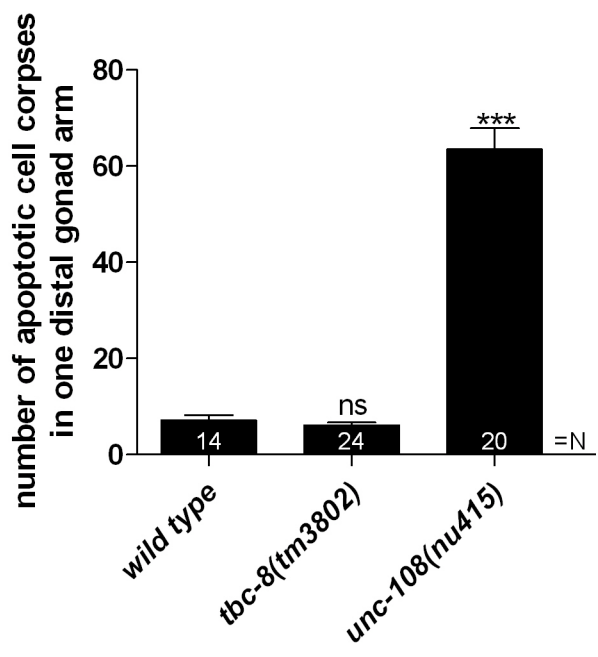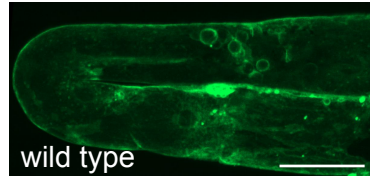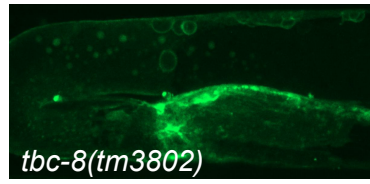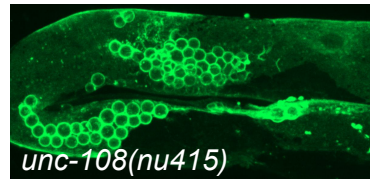

Supplement: Figure S6 — tbc-8(tm3802) mutants do not show defects in degradation of apoptotic cell corpses in the germ line. CED-1-GFP (bcIs39; [58]) was used as a marker to visualize apoptotic cell corpses in the germ line of C. elegans. Imaging stacks of one gonad arm were captured and the number of CED-1-GFP positive apoptotic cell corpses was recorded. tbc-8(tm3802) mutants displayed similar numbers of apoptotic cell corpses like wild type worms, whereas unc-108(nu415) mutants have defects in the engulfment of apoptotic cell corpses resulting in high numbers of corpses in their gonad arms, which was described previously [37], [38]. Scale bar represents 20 µm. Error bars = s.e.m. (***, P<0.0001; Student's t-test). (PDF) [file pgen.1002722.s006.pdf]

### NLP-21-derived VENUS fluorescence in dorsal nerve cord

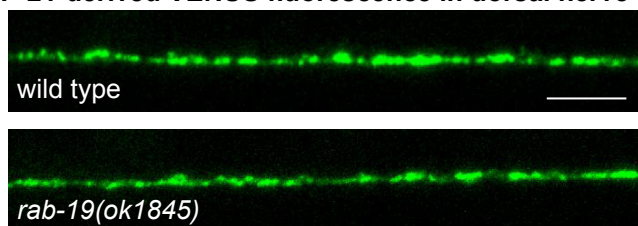

### NLP-21-derived VENUS in dorsal nerve cord

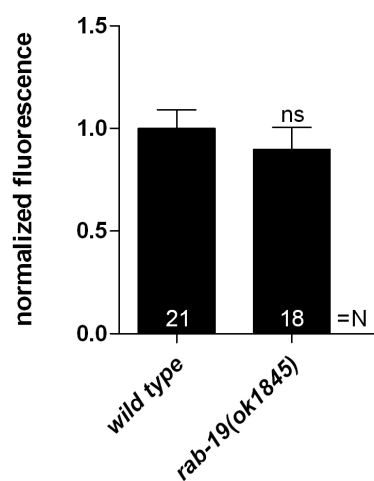

Supplement: Figure S7 — rab-19(ok1845) mutants do not display defects in DCV trafficking of the NLP-21-VENUS marker in the dorsal nerve cord. rab-19(ok1845) mutants have similar fluorescence levels of NLP-21-derived VENUS (89.83±10.69%) in the dorsal nerve cord like wild type worms. Scale bar represents 5 µm. Error bars = s.e.m. (ns, P>0.05; Student's t-test). (PDF) [file pgen.1002722.s007.pdf]

yeast two-hybrid

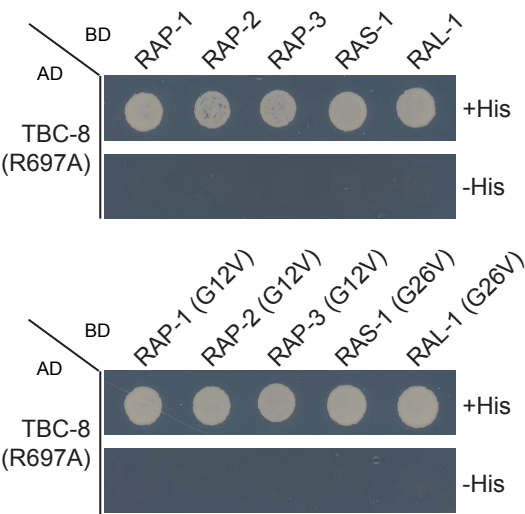

Supplement: Figure S8 — TBC-8 does not interact with RAP proteins in a yeast two-hybrid analysis. All three C. elegans RAP proteins, RAS-1 and RAL-1 in their native (upper panel) and their predicted activated state (lower panel) [59], [60], [61] were tested for interaction with TBC-8(R697A) in a yeast two-hybrid analysis. No growth on histidine-lacking plates was observed after 3 to 4 days. AD: Gal4p DNA activation domain fusion, BD: Gal4p DNA binding domain fusion, His: histidine. (PDF) [file pgen.1002722.s008.pdf]
