# Supplementary material for: TBC-8, a Putative RAB-2 GAP, Regulates Dense Core Vesicle Maturation in Caenorhabditis elegans
Source: PLoS Genet. 2012 May 24;8(5):e1002722. doi: 10.1371/journal.pgen.1002722 (PMC3359978; doi:10.1371/journal.pgen.1002722)
Supplement: Figure S4 — Protein sequence alignment of TBC-8 with its orthologs SGSM1 (H. sapiens) and CG32506-PC (D. melanogaster). Multiple sequence alignment was performed using the program MUSCLE and displayed using the program BOXSHADE. The predicted domains are color-coded. The RUN domain is shown in blue and the conserved blocks forming the ‘core’ of the RUN domain (A–F) are highlighted in black lines [35]. The TBC-domain (prediction made by the SMART program) is depicted in purple. The catalytic arginine residue, R697, is marked by a yellow arrowhead within its catalytic motif (yellow line). Note: domain lengths predicted for TBC-8 are shown. Accession number of SGSM1: NP_001035037; FlyBase ID of CG32506-PC: FBpp0300194. (PDF) [file pgen.1002722.s004.pdf]

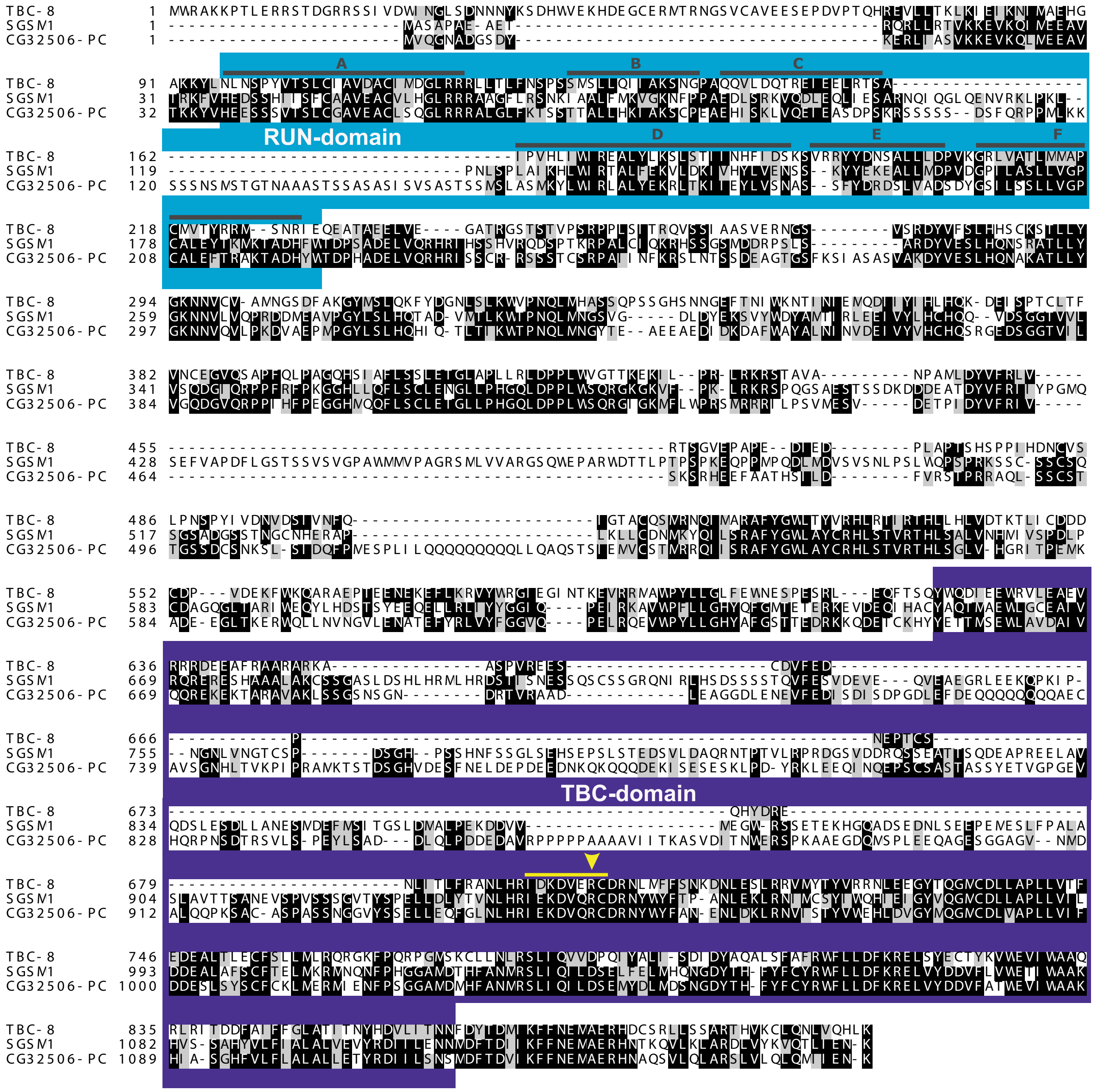
TBC-8
SGSM1
CG32506-PC
A
B
C
RUN-domain
D
E
F
TBC-domain
