# Supplementary material for: TBC-8, a Putative RAB-2 GAP, Regulates Dense Core Vesicle Maturation in Caenorhabditis elegans
Source: PLoS Genet. 2012 May 24;8(5):e1002722. doi: 10.1371/journal.pgen.1002722 (PMC3359978; doi:10.1371/journal.pgen.1002722)
Supplement: Table S1 — Strains used in this study. (PDF) [file pgen.1002722.s009.pdf]

Supplementary Table S1. Strains used in this study.

Strain name	Genotype
GQ669	*tbc-8(tm3802)*
MT1093	*unc-108(n501)*
KG1900	*unc-108(nu415)*
VC461	*egl-3(gk238)*
RB946	*ric-19(ok833)*
RB1537	*rab-19(ok1845)*
GQ688	*rab-19(ok1845);nuIs183[punc-129::nlp-21–venus]*
GQ584	*nuIs183[punc-129::nlp-21–venus]*
GQ026	*unc-108(n501);nuIs183[punc-129::nlp-21–venus]*
GQ142	*unc-108(nu415);nuIs183[punc-129::nlp-21–venus]*
GQ143	*egl-3(gk238);nuIs183[punc-129::nlp-21–venus]*
GQ641	*tbc-8(tm3802);nuIs183[punc-129::nlp-21–venus]*
GQ640	*ric-19(ok833);nuIs183[punc-129::nlp-21–venus]*
GQ642	*tbc-8(tm3802);unc-108(n501);nuIs183[punc-129::nlp-21–venus]*
GQ643	*tbc-8(tm3802);unc-108(nu415);nuIs183[punc-129::nlp-21–venus]*
GQ644	*tbc-8(tm3802);egl-3(gk238);nuIs183[punc-129::nlp-21–venus]*
GQ645	*tbc-8(tm3802);ric-19(ok833);nuIs183[punc-129::nlp-21–venus]*
GQ646	*tbc-8(tm3802);nuIs183;gzEx195[prab-3::tagRFPt-tbc-8]*
GQ647	*tbc-8(tm3802);nuIs183;gzEx196[punc-129::tagRFPt-tbc-8]*
GQ648	*tbc-8(tm3802);nuIs183;gzEx197[punc-129::tagRFPt-tbc-8(R697A)]*
GQ649	*tbc-8(tm3802); nuIs183;gzEx198[prab-3::mcherry-rab-5(Q78L)]*
GQ585	*nuIs195[punc-129::ins-22–venus]*
GQ650	*tbc-8(tm3802);nuIs195[punc-129::ins-22–venus]*
GQ586	*ceIs72[punc-129::ida-1–gfp]*
GQ651	*tbc-8(tm3802);ceIs72[punc-129::ida-1–gfp]*
KG1645	*ceIs61[punc-129::flp-3–venus]*
GQ652	*tbc-8(tm3802);ceIs61[punc-129::flp-3–venus]*
KP3292	*nuIs152[punc-129::gfp–snb-1]*
GQ653	*tbc-8(tm3802);nuIs152[punc-129::gfp–snb-1]*
KP3931	*nuIs168[punc-129::venus–rab-3]*
GQ654	*tbc-8(tm3802);nuIs168[punc-129::venus–rab-3]*
Bristol	*N2 (wild type)*
GQ655	*N2;gzEx199[ptbc-8::gfp]*
GQ661	*N2;gzEx205[prab-3::yfp–tbc-8; prab-3::mcherry–rab-2]*
GQ662	*N2;gzEx206[prab-3::tagRFPt–tbc-8; prab-3::manns–yfp]*
GQ663	*N2;gzEx207[prab-3::yfp–tbc-8; prab-3::mcherry–apt-9]*
GQ664	*N2;gzEx208[prab-3::yfp–tbc-8; prab-3::mcherry–rab-5]*
GQ665	*N2;gzEx209[prab-3::yfp–tbc-8; prab-3::mcherry–rab-7]*
GQ666	*N2;gzEx210[prab-3::ric-19–yfp]*
GQ703	*N2;gzEx213[prab-3::tagRFPt–tbc-8; prab-3::ric-19–yfp]*
GQ704	*unc-108(nu415);gzEx213[prab-3::tagRFPt–tbc-8; prab-3::ric-19–yfp]*
GQ667	*tbc-8(tm3802);gzEx210[prab-3::ric-19–yfp]*
DH1336	*bIs34[*prme-8::rme-8-gfp; rol-6(su1006)]*
GQ672	*tbc-8(tm3802);bIs34[*prme-8::rme-8-gfp; rol-6(su1006)]*
GS1826	*arIs36[*phsp::ssgfp]*
GQ673	*tbc-8(tm3802);arIs36[*phsp::ssgfp]*

GS2526	*arIs37[*p*myo-3::ssgfp]*
GQ674	*tbc-8(tm3802);arIs37[*p*myo-3::ssgfp]*
MD701	*bcIs39[*p*lim-7::ced-1-gfp]*
GQ675	*tbc-8(tm3802);bcIs39[*p*lim-7::ced-1-gfp]*
GQ676	*unc-108(nu415);bcIs39[*p*lim-7::ced-1-gfp]*
GQ677	*eri-1(mg366);nuIs183[punc-129::nlp-21–venus]*
GQ678	*tbc-1(tm2282)*
GQ679	*tbc-1(tm2282);nuIs183[punc-129::nlp-21–venus]*
XW1069	*tbc-2(qx20)*
GQ680	*tbc-2(qx20);nuIs183[punc-129::nlp-21–venus]*
GQ681	*tbc-4(tm3255)*
GQ682	*tbc-4(tm3255);nuIs183[punc-129::nlp-21–venus]*
RB1959	*tbc-11(ok2576)*
GQ683	*tbc-11(ok2576);nuIs183[punc-129::nlp-21–venus]*
VC821	*tbc-12(gk362)*
GQ684	*tbc-12(gk362);nuIs183[punc-129::nlp-21–venus]*
RB1516	*tbc-13(ok1812)*
GQ685	*tbc-13(ok1812);nuIs183[punc-129::nlp-21–venus]*
GQ686	*tbc-18(ok2374)*
GQ687	*tbc-18(ok2374);nuIs183[punc-129::nlp-21–venus]*
