# Supplementary material for: TBC-8, a Putative RAB-2 GAP, Regulates Dense Core Vesicle Maturation in Caenorhabditis elegans
Source: PLoS Genet. 2012 May 24;8(5):e1002722. doi: 10.1371/journal.pgen.1002722 (PMC3359978; doi:10.1371/journal.pgen.1002722)
Supplement: Table S2 — Transgenic arrays used in this assay. (PDF) [file pgen.1002722.s010.pdf]

Supplementary Table S2. Transgenic arrays used in this assay.

**Array name**	**Plasmid**	**Markers**
gzEx195	20 ng/µl *prab-3::tagRFPt–tbc-8*	20 ng/µl *pttx-3::gfp*
gzEx196	20 ng/µl *punc-129::tagRFPt–tbc-8*	20 ng/µl *pttx-3::gfp*
gzEx197	20 ng/µl *punc-129::tagRFPt–tbc-8(R697A)*	20 ng/µl *pttx-3::gfp*
gzEx198	5 ng/µl *prab-3::mcherry–rab-5(Q78L)*	20 ng/µl *pttx-3::gfp*
gzEx199	15 ng/µl *ptbc-8::gfp*	40 ng/µl *pRF4-rol-6(su1006)*
gzEx205	20 ng/µ*l prab-3::yfp–tbc-8*	40 ng/µl *pRF4-rol-6(su1006)*
	5 ng/µl *prab-3::mcherry–rab-2*	20 ng/µl *pttx-3::gfp*
gzEx206	20 ng/µl *prab-3::tagRFPt–tbc-8;*	40 ng/µl *pRF4-rol-6(su1006)*
	10 ng/µl *prab-3::manns–yfp*	20 ng/µl *pttx-3::gfp*
gzEx207	20 ng/µl *prab-3::yfp–tbc-8;*	40 ng/µl *pRF4-rol-6(su1006)*
	10 ng/µl *prab-3::mcherry–apt-9*	20 ng/µl *pttx-3::gfp*
gzEx208	20 ng/µl *prab-3::yfp–tbc-8;*	40 ng/µl *pRF4-rol-6(su1006)*
	5 ng/µl *prab-3::mcherry–rab-5*	20 ng/µl *pttx-3::gfp*
gzEx209	20 ng/µl *prab-3::yfp–tbc-8;*	40 ng/µl *pRF4-rol-6(su1006)*
	5 ng/µl *prab-3::mcherry–rab-7*	20 ng/µl *pttx-3::gfp*
gzEx210	20 ng/µl *prab-3::ric-19–yfp*	40 ng/µl *pRF4-rol-6(su1006)*
		20 ng/µl *pttx-3::gfp*
		20 ng/µl *punc-129::tagRFP*
gzEx213	20 ng/µl *prab-3::tagRFPt–tbc-8*	40 ng/µl *pRF4-rol-6(su1006)*
	20 ng/µl *prab-3::ric-19–yfp*	20 ng/µl *pttx-3::gfp*
