# Supplementary material for: TBC-8, a Putative RAB-2 GAP, Regulates Dense Core Vesicle Maturation in Caenorhabditis elegans
Source: PLoS Genet. 2012 May 24;8(5):e1002722. doi: 10.1371/journal.pgen.1002722 (PMC3359978; doi:10.1371/journal.pgen.1002722)
Supplement: Table S3 — Constructs used in this study. (PDF) [file pgen.1002722.s011.pdf]

Supplementary Table S3. Constructs used in this study:

prab-3::tagRFPt–tbc-8
punc-129::tagRFPt–tbc-8
punc-129::tagRFPt–tbc-8(R697A)
ptbc-8::gfp
prab-3::yfp–tbc-8
prab-3::mcherry–rab-2
prab-3::tagRFPt–tbc-8
prab-3::manns–yfp
prab-3::mcherry–apt-9
prab-3::mcherry–rab-5
prab-3::mcherry–rab-5(Q78L)
prab-3::mcherry–rab-7
prab-3::ric-19–yfp
punc-129::tagRFP
GADT7_*tbc-8*
GADT7_*tbc-8(R697A)*
GADT7_*ica69*
GADT7_*ric-19*
GBKT7_*rab-1(Q70L)*
GBKT7_*rab-2 (Q65L)*
GBKT7_*rab-3(Q81L)*
GBKT7_*rab-5(Q78L)*
GBKT7_*rab-6.1(Q70L)*
GBKT7_*rab-6.2(Q69L)*
GBKT7_*rab-7(Q68L)*
GBKT7_*rab-8(Q67L)*
GBKT7_*rab-10(Q68L)*
GBKT7_*rab-11.1(Q70L)*
GBKT7_ *rab-14(Q70L)*
GBKT7_*rab-18(Q70L)*
GBKT7_*rab-19(Q69L)*
GBKT7_*rab-21(Q71L)*
GBKT7_*rab-28(Q95L)*
GBKT7_*rab-30(Q66L)*
GBKT7_*rab-33(Q159L)*
GBKT7_*rab-35(Q69L)*
GBKT7_*rab-37.1(Q37L)*
GBKT7_*rab-37.2(Q89L)*
GBKT7_*rab-39(Q77L)*
GBKT7_*rab-2(S20N)*
GBKT7_*rab-19(T24N)*
GBKT7_*rab-14(S25N)*
GBKT7_*rap-1*
GBKT7_*rap-2*
GBKT7_*rap-3*
GBKT7_*ras-1*
GBKT7_*ral-1*
GBKT7_*rap-1(G12V)*
GBKT7_*rap-2(G12V)*

GBKT7_*rap-3(G12V)*
GBKT7_*ras-1(G26V)*
GBKT7_*ral-1(G26V)*
L4440
L4440_*tbc-8*
pcDNA6.2_*EmGFP*_Dest
pcDNA6.2_*EmGFP-ric-19*
pcDNA3.1_*V5–tbc-8*
pcDNA3.1_*V5–tbc-8(1-597 aa)*
